# Epidemiology of acute hepatitis C and hepatitis C virus-related cirrhosis in reproductive-age women, 1990–2019: An analysis of the Global Burden of Disease study

**DOI:** 10.7189/jogh.14.04077

**Published:** 2024-04-19

**Authors:** Yanzheng Zou, Ming Yue, Xiangyu Ye, Yifan Wang, Xinyan Ma, Amei Zhang, Xueshan Xia, Hongbo Chen, Rongbin Yu, Sheng Yang, Peng Huang

**Affiliations:** 1 Department of Epidemiology, National Vaccine Innovation Platform, Centre for Global Health, School of Public Health, Nanjing Medical University, Nanjing, China; 2 Department of Infectious Diseases, The First Affiliated Hospital of Nanjing Medical University, Nanjing, China; 3 Department of Infectious Disease, Jurong Hospital Affiliated to Jiangsu University, Jurong, China; 4 Faculty of Life Science and Technology, Kunming University of Science and Technology, Kunming, China; 5 Kunming Medical University, Kunming, China; 6 Department of Biostatistics, National Vaccine Innovation Platform, Centre for Global Health, School of Public Health, Nanjing Medical University, Nanjing, China

## Abstract

**Background:**

The current study uniquely focuses on the global incidence and temporal trends of acute hepatitis C (AHC) and hepatitis C virus (HCV)-related cirrhosis among women of reproductive age (15–49 years) from 1990–2019. The risk of vertical transmission and adverse perinatal outcomes associated with HCV infection underscores the importance of prioritising these women in HCV prevention efforts.

**Methods:**

Leveraging the Global Burden of Disease 2019 data, we calculated age-standardised incidence rates (ASIR) and assessed temporal trends via the average annual percent change from joinpoint regression. The age-period-cohort model was employed to understand further the effects of age, period, and birth cohort.

**Results:**

Over the 30 years, global incidences of AHC and HCV-related cirrhosis in reproductive-age women increased by 46.45 and 72.74%, respectively. The ASIR of AHC was highest in low sociodemographic index regions but showed a declining trend. Conversely, the ASIR of HCV-related cirrhosis displayed unfavourable trends in low, low-middle, and high sociodemographic index regions. Special attention is necessary for sub-Saharan Africa, high-income North America, Eastern Europe, and Central Asia due to their high incidence rates or increasing trends of AHC and HCV-related cirrhosis. Notably, the age-period-cohort model suggests a recent resurgence in AHC and HCV-related cirrhosis risk.

**Conclusions:**

The current study is the first to thoroughly evaluate the trends of AHC and HCV-related cirrhosis among reproductive-age women, shedding light on previously unexplored aspects of HCV epidemiology. Our findings identify critical areas where health care systems must adapt to the changing dynamics of HCV infection. The detailed stratification by region and nation further enables the development of localised prevention and treatment strategies.

Hepatitis C virus (HCV) infection remains a serious global health concern, with an estimated 58 million individuals chronically infected worldwide and approximately 1.5 million new infections annually [1]. In response to the substantial global health burden posed by viral hepatitis, the World Health Organization (WHO) initiated a proposal to eliminate hepatitis C as a public health threat by 2030 [2]. However, the majority of HCV research has focused on the general population or certain high-risk subgroups, such as individuals co-infected with human immunodeficiency virus or people who inject drugs [3,4]. Other key populations, such as women of reproductive age (15–49 years), are frequently underrepresented in these studies. This research gap is partly because routine HCV screening, which is common in high-risk groups, is not standard for reproductive-age women, resulting in many HCV infections going undetected. The lack of data limits research efforts to understand the true burden of HCV in this population.

Representing almost a quarter of the global population, the health of reproductive-age women is intertwined with the well-being of their offspring. HCV-infected pregnant women face a 5% risk of vertical transmission, with no current preventive measures available [5]. The use of direct-acting antivirals (DAAs) – effective in curing over 95% of HCV cases – is not recommended during pregnancy due to insufficient safety evidence [6]. Moreover, previous studies have indicated that women who have ever been infected with HCV, past or present, face increased risks of adverse maternal and foetal outcomes, including intrahepatic cholestasis of pregnancy, preterm birth, and small for gestational age infants [7,8]. The presence of liver cirrhosis further exacerbates the risks, leading to an even higher likelihood of unfavourable health outcomes for both the pregnant women and their foetuses [9]. Given the risk of vertical transmission and the associated adverse perinatal outcomes of HCV infection during pregnancy, early prevention, detection, and treatment are critical in women of reproductive age, ideally before pregnancy.

In 2019, an estimated 15 million women of reproductive age were affected by HCV worldwide, corresponding to a prevalence of 0.78% [10]. Alarmingly, the incidence of HCV infection among reproductive-age women is on the rise in many regions globally. In the United States, for instance, reported cases of acute hepatitis C (AHC) increased 3.4-fold among reproductive-age women between 2006–14 [11]. While these previous studies have provided valuable insights into aspects of HCV epidemiology in reproductive-age women, they either did not explore the temporal trends of HCV infection or were limited to situations in a single country. Additionally, a notable gap exists in studies regarding HCV-related cirrhosis among reproductive-age women. Our study seeks to fill these gaps with a comprehensive and global analysis of the epidemiology of AHC and HCV-related cirrhosis in this demographic.

Understanding the epidemiology of AHC and HCV-related cirrhosis among reproductive-age women is crucial for achieving the WHO’s goal to eliminate HCV by 2030. The key to this goal is to reduce the incidence of new HCV infections by 90%. By identifying and treating HCV in reproductive-age women, we can significantly disrupt vertical transmission to the next generation, thereby contributing to the reduction of new infections. In addition, in-depth knowledge of HCV trends in this demographic is essential for developing informed public health policies and targeted treatment strategies. These efforts align with the WHO’s additional targets of treating 80% of eligible HCV patients and reducing liver-related mortality by 65% [12].

In summary, the present study sets out to evaluate the geographical and temporal trends in the incidence of AHC and HCV-related cirrhosis among reproductive-age women from 1990–2019, using data from the Global Burden of Disease (GBD) 2019 database. Our study also employs the age-period-cohort (APC) model to assess the influence of recent health policies and emerging risk factors, offering a comprehensive insight into temporal trends.

## METHODS

### Study data

This study utilised data from the GBD 2019 study, which provided estimates of epidemiologic assessments of 256 causes of death, 369 diseases and injuries, and 87 risk factors from 204 countries and territories [13]. Details and estimation methodologies of the GBD 2019 study have been published previously [14]. Based on the WHO definition, we defined women of reproductive age as those aged between 15–49 years. We obtained data from the Global Health Data Exchange on estimates of the incidence number and rates of AHC and HCV-related cirrhosis for this demographic.

The GBD study collected and compiled epidemiological data from various sources, including hospital databases, health system records, and published studies. This data was then standardised and mapped onto the corresponding International Classification of Diseases (ICD) codes. The mapping process involved aligning the epidemiological data with the ICD codes to ensure that each incidence was accurately categorised. The ICD-10 codes for AHC are B17.1–B17.11, B18.2, and B19.2–B19.21 [15]. However, while the ICD-10 code is sufficient for defining liver cirrhosis, it does not specify the underlying aetiology. To address this, the GBD study used a systematic literature review and various adjusting models to split cirrhosis incidence into specific causes: hepatitis B, hepatitis C, non-alcoholic fatty liver disease, alcohol-related liver diseases, and others. The literature review included published case series that reported the proportion of cases due to these aetiologies. These proportions were then applied to cirrhosis estimates at the draw level to calculate aetiology-specific incidence rates. The detailed methods of processing and modelling these proportion data can be found in previously published literature [16].

DisMod-MR 2.1 (Institute for Health Metrics and Evaluation, University of Washington, Seattle, WA, USA), a Bayesian meta-regression tool, was used to pool the incidence of AHC and HCV-related cirrhosis and generate age-sex-location-year-specific estimates. All GBD estimates come with 95% uncertainty intervals (UIs). The 95% UIs represent the 2.5th–97.5th percentile range based on the distribution of 1000 draws at each GBD estimation step, indicating the uncertainty propagated through each step [17]. Scalars derived from the Healthcare Access and Quality Index were used to adjust for discrepancies in incidence rates caused by incomplete health care access in populations where not all individuals with a disease might be captured by health system records [18].

### Sociodemographic index and geographic regions

The socioeconomic development status of the countries and territories was determined using the sociodemographic index (SDI). The SDI is a composite measure comprising the total fertility rate in women under 25 years, mean education for individuals aged 15 and older, and lag-distributed income per person. It is important to note that while the SDI includes the total fertility rate for women under 25 years, this component is utilised as a proxy for women’s societal status, particularly reflecting the impact of early childbearing on education and workforce participation. Our focus was on exploring the broader influence of socioeconomic factors, as represented by the SDI, on health outcomes rather than on fertility rates themselves. The detailed methodology for generating SDI has been described elsewhere [19]. The SDI value ranges from zero to one, where a higher value indicates a more advanced socioeconomic status. According to the SDI values from 2019, 204 countries and territories were divided into five categories – high, high-middle, middle, low-middle, and low. Additionally, these locations were grouped into 21 GBD regions based on their geographical areas.

### Statistical analysis

To normalise age structure variations among populations, we calculated the age-standardised incidence rates (ASIR) and the corresponding 95% UIs based on the world standard population reported in the GBD 2019 study [20]. We used Monte Carlo simulation to propagate uncertainties, creating 1000 modelled instances for each incidence [21]. The 95% UIs were then defined by the 2.5th and 97.5th percentiles.

Temporal trends in ASIR from 1990–2019 were assessed by calculating the average annual percent change (AAPC) with the corresponding 95% confidence intervals (CIs) using joinpoint regression [22]. The Joinpoint Regression Program starts with the minimum number of joinpoints and tests whether additional joinpoints are statistically significant and must be added to the model. For this study, the number of joinpoints was determined through the grid search method, and the Monte Carlo permutation method was employed in the test for significance. The AAPC was then calculated from the annual percent change of each segment within the final significant model. An AAPC with a lower 95% CI above zero indicates increasing ASIR, while an AAPC with an upper 95% CI below zero signifies decreasing ASIR. If the 95% CI of the AAPC includes zero, the ASIR is stable.

The web tool provided by the United States National Cancer Institute (http://analysistools.nci.nih.gov/apc/) was used to perform the APC analysis in the current study. The web tool was implemented with estimable APC functions and corresponding Wald tests in R codes [23]. For input data, the age-specific incidence rates for five-year age intervals, ranging from 15–19 years to 45–49 years, were extracted from the GBD 2019 data. In addition, the data were rearranged into successive five-year periods: from 1990–94 to 2015–19, with 2000–04 as the reference period; also, the population was grouped into 12 overlapping ten-year birth cohorts from 1940–49 (the 1945 cohort) to 1995–2004 (the 2000 cohort) as referenced by the 1965–74 (the 1970 cohort) birth cohort.

The APC model output includes net drift, local drift, age, period, and cohort effects. Net drift represents the overall annual percentage change in incidence rate while accounting for period and cohort changes. Local drift, on the other hand, represents the age-specific annual percentage changes, indicating variations in trends across different age groups. A net or local drift above zero is regarded as an upward trend, while a value below zero indicates a downward trend. The Wald χ2 test was used to test for the significance of the trend. The longitudinal age curve depicts the age effect, which is the fitted longitudinal age-specific rates in reference cohorts adjusted for period deviations. The period effect refers to the relative risk (RR) of each period compared to the reference period. The cohort effect refers to the RR of each cohort compared to the reference cohort [24].

Lastly, to explore factors influencing ASIR and AAPC, the Pearson correlation was applied to examine the correlation between 1) SDI and ASIR in 2019, 2) SDI and AAPC, as well as 3) AAPC and ASIR in 1990 at the national level. A two-tailed *P*-value <0.05 was considered statistically significant. Statistical analysis was performed with the Joinpoint Regression Program software, version 4.9.0.0 (National Cancer Institute, Bethesda, Maryland, USA), and R software, version 4.1.2 (R Core Team, Vienna, Austria).

## RESULTS

### Incidence of AHC and HCV-related cirrhosis among women of reproductive age (2019)

Globally, there were 856 953 (95% UI = 629 470, 1 136 117) incidence cases of AHC and 133 881 (95% UI = 91 540, 186 809) incidence cases of HCV-related cirrhosis among women of reproductive age in 2019. The age-standardised incidence rate per 100 000 population for AHC and HCV-related cirrhosis was 43.9 (95% UI = 37.02, 56.02) and 6.64 (95% UI = 5.10, 9.50), respectively.

By SDI category, the highest ASIR of AHC among women of reproductive age was found in the low SDI region (ASIR = 74.32; 95% UI = 62.25, 96.04). Unexpectedly, the highest ASIR of HCV-related cirrhosis was found in the high SDI region (ASIR = 12.13; 95% UI = 9.78, 15.60), where the disease incidence is usually low. At the regional level, central sub-Saharan Africa had the highest ASIR of AHC (ASIR = 152.1; 95% UI = 127.74, 197.93), followed by North Africa. Regarding HCV-related cirrhosis, the highest ASIR was found in high-income North America (ASIR = 20.78; 95% UI = 16.64, 26.35), followed by central Latin America (**Table 1**, **Table 2**).

**Table 1:** Incidence cases and age-standardised incidence rate of acute hepatitis C in women of reproductive age in 1990 and 2019, and its temporal trends, by SDI categories and GBD regions

	Incidence cases				Age-standardised incidence rate		
**Characteristics**	**Cases in 1990 (95% UI)**		**Cases in 2019 (95% UI)**	**Percentage change (%)**	**ASIRs per 100 000 in 1990 (95% UI)**	**ASIRs per 100 000 in 2019 (95% UI)**	**AAPC (95% CI)**
Global	585 159.6 (427 343.8, 782 548.4)		856 952.9 (629 470.1, 113 611.7)	46.45	44.19 (37.62, 57.37)	43.9 (37.02, 56.02)	–0.02 (–0.06, 0.03)
Sociodemographic index							
*Low SDI*	94 171.2 (69 035.7, 126 135.9)		193 873.6 (144 077.7, 257 311.7)	105.87	83.64 (72.25, 108.19)	74.32 (62.25, 96.04)	–0.43 (–0.57, –0.29)
*Low-middle SDI*	121 411.4 (88 861.3, 163 363)		199 699.3 (146 146.5, 267 306.5)	64.48	46.3 (39.42, 59.13)	43.3 (36.55, 55.05)	–0.24 (–0.29, –0.19)
*Middle SDI*	201 502.1 (147 705.4, 270 808.4)		279 860.8 (207 365.4, 366 410.3)	38.89	45.94 (39.05, 59.47)	44.36 (38.27, 56.12)	–0.1 (–0.23, 0.03)
*High-middle SDI*	104 956.9 (75 980.9, 141 990.1)		107 887.5 (77 517.5, 146 297.6)	2.79	35.52 (29.60, 45.15)	30.38 (26.11, 38.99)	–0.51 l (–0.58, –0.43)
*High SDI*	62 724.2 (45 643.7, 81 940.8)		75 021 (54 872.0, 98 836.9)	19.60	29.3 (24.81, 37.84)	31.7 (26.63, 39.67)	0.29 (0.16, 0.42)
Region							
*Andean Latin America*	4040.1 (2946.1, 5352.3)		6400.0 (4633.5, 8402.6)	89.36	43.52 (36.95, 55.89)	38.66 (33.34, 50.21)	–0.42 (–0.46, –0.37)
*Australasia*	1878.3 (1334.1, 2607.0)		2579.8 (1784.3, 3503.9)	2967.05	34.9 (29.33, 46.11)	36.76 (31.79, 48.51)	0.18 (0.13, 0.23)
*Caribbean*	6288.6 (4617.2, 8345.7)		7650.5 (5627.8, 10163.8)	–49.65	68.28 (58.46, 87.98)	63.32 (54.10, 81.62)	–0.26 (–0.31, –0.22)
*Central Asia*	10 225 (7175.9, 14 312.7)		16 329.5 (11 410.9, 22 863.5)	59.70	60.49 (52.41, 79.42)	67.99 (57.48, 90.41)	0.41 (0.39, 0.43)
*Central Europe*	7541.3 (5274.7, 10 456.9)		5849.3 (4096.6, 8172.0)	–22.44	25.05 (21.17, 32.83)	22.57 (19.40, 29.58)	–0.37 (–0.41, –0.33)
*Central Latin America*	37 917.7 (28 944.9, 49 377.5)		52 435.6 (39 899.7, 68 238.2)	–93.20	89.67 (76.71, 114.43)	77.73 (65.43, 96.22)	–0.5 (–0.70, –0.31)
*Central sub-Saharan Africa*	19 915.2 (14 610.4, 27 046.3)		43 970.1 (32 526.7, 58 344.0)	120.79	174.44 (145.53, 224.37)	152.1 (127.74, 197.93)	–0.48 (–0.49, –0.46)
*East Asia*	75 458.7 (50 277, 109 918.9)		47 240.9 (30 578.5, 70 671.6)	–37.40	21.59 (18.08, 29.77)	13.78 (11.61, 19.20)	–1.48 (–1.72, –1.24)
*Eastern Europe*	20 743.2 (14 511.5, 28 480.8)		22 476.7 (15 805, 31 366.9)	8.36	37.86 (32.68, 50.16)	46.83 (40.58, 61.02)	0.75 (0.70, 0.80)
*Eastern sub-Saharan Africa*	35 413.0 (25 898.0, 46 835.6)		70 128.6 (52 451.4, 92 251.1)	364.75	87.62 (73.87, 111.35)	73.1 (61.47, 94.66)	–0.64 (–0.72, –0.56)
*High-income Asia Pacific*	12 237.6 (9042.2, 15 802.0)		7131.0 (5007.3, 9540.1)	128.24	25.59 (21.51, 33.19)	15.61 (13.09, 19.98)	–1.71 (–2.33, –1.09)
*High-income North America*	21 672.1 (15 266.4, 29 020)		27 931.3 (20 230.8, 36 775.6)	–70.47	29.04 (24.77, 37.61)	33.1 (27.6, 42.88)	0.51 (0.38, 0.65)
*North Africa and the Middle East*	79 326 (58 714.5, 104 509.1)		164 583.3 (126 306.8, 209 022.2)	–63.85	111.87 (94.80, 144.80)	104.61 (88.52, 132.19)	–0.24 (–0.28, –0.19)
*Oceania*	249.2 (167.8, 364.3)		490.6 (331.6, 716.5)	96.87	15.82 (13.51, 21.25)	14.62 (12.48, 19.78)	–0.27 (–0.31, –0.24)
*South Asia*	70 804.5 (49 662.5, 96 701.5)		110 083.6 (77 053.4, 151 204.1)	54.36	28.88 (24.40, 38.33)	23.46 (20.30, 31.02)	–0.73 (–0.81, –0.66)
*Southeast Asia*	45 888.5 (32 279, 63 033.1)		59 448.3 (42 486.1, 79 971)	29.55	36.95 (31.72, 49.10)	33.34 (28.71, 43.06)	–0.35 (–0.39, –0.31)
*Southern Latin America*	2504.0 (1857.8, 3240.5)		3166.5 (2281.8, 4132.7)	1994.07	20.46 (17.41, 26.20)	18.06 (15.20, 23.14)	–0.41 (–0.50, –0.32)
*Southern sub-Saharan Africa*	8153.4 (5933.4, 10 850.2)		13 478.1 (9765.9, 18 207.8)	65.31	63.5 (54.14, 81.79)	64.39 (54.46, 83.66)	0.05 (–0.02, 0.12)
*Tropical Latin America*	39 197.6 (28 594.2, 51 846.7)		57 608.4 (41 986.2, 76 219.9)	180.84	99.22 (84.47, 127.32)	95.18 (81.67, 121.03)	–0.21 (–0.58, 0.16)
*Western Europe*	33 594.4 (25 570.6, 42 577.3)		28 679.0 (21 375.1, 37 352.0)	–78.77	34.54 (29.19, 43.13)	28.51 (24.38, 36.09)	–0.66 (–0.69, –0.63)
*Western sub-Saharan Africa*	52 111.3 (38 177.8, 70 238.5)		109 291.9 (80 939.5, 145 870.3)	34.57	122.57 (105.70, 156.45)	100.18 (85.73, 129.76)	–0.70 (–0.75, –0.64)

AAPC – average annual percent change, ASIR – age-standardised incidence rate, CI – confidence interval, GBD – Global Burden of Disease, SDI – sociodemographic index, UI – uncertainty interval

**Table 2:** Incidence cases and age-standardised incidence rate of cirrhosis due to hepatitis C in women of reproductive age in 1990 and 2019, and its temporal trends, by SDI categories and GBD regions

	Incidence cases				Age-standardised incidence rate		
**Characteristics**	**Cases in 1990 (95% UI)**		**Cases in 2019 (95% UI)**	**Percentage change (%)**	**ASIRs per 100 000 in 1990 (95% UI)**	**ASIRs per 100 000 in 2019 (95% UI)**	**AAPC (95% CI)**
**Global**	77 503.8 (51 236.7, 109 393.1)		133 881.3 (91 540.2, 186 808.9)	72.74	6.58 (5.02, 9.54)	6.64 (5.10, 9.50)	0.03 (–0.05, 0.11)
Sociodemographic index							
*Low SDI*	4491.4 (2450.8, 6934.9)		12 277.3 (7227.0, 18 748.5)	173.35	4.80 (3.34, 7.77)	5.56 (3.81, 8.83)	0.49 (0.46, 0.52)
*Low-middle SDI*	9287.8 (5446.3, 13 955.3)		23 473.4 (14 102.7, 35 371.9)	152.73	4.26 (3.07, 6.59)	5.39 (3.84, 8.15)	0.80 (0.77, 0.83)
*Middle SDI*	23 095.4 (14 550.1, 33 656.3)		41 602.5 (26 698.1, 59 462.6)	80.13	6.39 (4.49, 9.41)	6.16 (4.65, 8.95)	–0.12 (–0.15, –0.08)
*High-middle SDI*	17 791.3 (12 164.8, 25 017.5)		24 254.3 (16 354.3, 34 057.8)	36.33	6.39 (5.00, 8.97)	5.77 (4.39, 8.25)	–0.36 (–0.42, –0.29)
*High SDI*	22 805.9 (16 690.0, 30 171.4)		32 216.3 (24 411.0, 40 775.6)	41.26	10.20 (8.33, 13.61)	12.13 (9.78, 15.60)	0.62 (0.49, 0.76)
Region							
*Andean Latin America*	183.4 (103.8, 296.3)		486.1 (273.2, 806.9)	165.05	2.49 (1.88, 4.01)	2.98 (2.22, 4.71)	0.62 (0.56, 0.67)
*Australasia*	268.2 (191.7, 353.9)		404 (290.0, 530.5)	730.35	4.85 (3.83, 6.69)	5.31 (4.20, 7.31)	0.28 (0.13, 0.44)
*Caribbean*	319.4 (187.7, 482.1)		539.9 (315.2, 832.1)	2016.69	4.05 (3.11, 6.28)	4.37 (3.26, 6.74)	0.26 (0.19, 0.34)
*Central Asia*	804.1 (486.8, 1221.9)		2668.1 (1587.3, 4151.8)	231.81	6.05 (4.36, 9.15)	10.85 (8.05, 16.47)	2.02 (1.96, 2.08)
*Central Europe*	2233.6 (1461.0, 3213.8)		1973.8 (1271.6, 2838.9)	–11.63	6.89 (5.46, 9.61)	6.05 (4.64, 8.61)	–0.45 (–0.50, –0.41)
*Central Latin America*	4543.2 (3122.9, 6217.2)		8344 (5473.2, 11 641)	83.66	14.20 (10.72, 19.64)	12.23 (9.18, 17.25)	–0.47 (–0.60, –0.34)
*Central sub-Saharan Africa*	593.0 (333.2, 921.6)		1877.3 (1156.8, 2781.8)	216.58	5.97 (4.26, 10.02)	7.30 (5.30, 11.41)	0.74 (0.62, 0.86)
*East Asia*	17 708.6 (10 845.6, 26 325.8)		25 935.4 (16 872.6, 37 050.4)	46.46	6.30 (4.58, 9.45)	5.75 (4.44, 8.18)	–0.30 (–0.37, –0.24)
*Eastern Europe*	1906.9 (1200.4, 2823.1)		3221.8 (1862.9, 4951.0)	68.95	3.27 (2.43, 4.86)	5.43 (4.16, 8.56)	1.77 (1.70, –1.84)
*Eastern sub-Saharan Africa*	1899.9 (1111.3, 2870.9)		4659.2 (2830.1, 7071.3)	145.23	5.82 (3.71, 9.49)	5.86 (4.25, 9.01)	0.03 (–0.03, 0.08)
*High-income Asia Pacific*	6869.1 (4516.8, 9366.2)		6189.2 (4470.9, 8033.3)	–92.14	13.66 (10.52, 18.73)	12.13 (9.51, 16.26)	–0.40 (–0.53, –0.27)
*High-income North America*	10 411.5 (7457.0, 14 005.5)		19 067.3 (14 768.2, 23 845.2)	6.24	13.10 (10.37, 17.6)	20.78 (16.64, 26.35)	1.59 (1.42, 1.75)
*North Africa and the Middle East*	3324.1 (2040.4, 4881.4)		11 061.4 (6897.5, 16 064.6)	509.58	5.50 (3.78, 8.34)	7.16 (5.16, 10.6)	0.93 (0.79, 1.08)
*Oceania*	18.9 (11.0, 30.1)		39.2 (22.3, 61.8)	107.41	1.44 (1.10, 2.39)	1.25 (0.96, 2.02)	–0.47 (–0.52, –0.41)
*South Asia*	6385.4 (3022.9, 10 342.6)		20 263.1 (10 794.4, 32 400.1)	–3.07	2.98 (1.98, 5.00)	4.51 (3.04, 7.16)	1.41 (1.34, 1.48)
*Southeast Asia*	7149.4 (4133.2, 10 830.5)		12 344.2 (7827.9, 17 957.6)	72.66	7.10 (4.95, 10.92)	6.60 (4.81, 9.81)	–0.26 (–0.31, –0.22)
*Southern Latin America*	940 (622.4, 1331.1)		1519.8 (995.1, 2170.9)	61.68	7.89 (5.98, 11.01)	8.31 (6.41, 11.94)	0.18 (0.13, 0.24)
*Southern sub-Saharan Africa*	406.7 (225.3, 646.0)		700.2 (394.1, 1100.9)	72.17	3.53 (2.50, 5.61)	3.39 (2.46, 5.39)	–0.15 (–0.20, –0.09)
*Tropical Latin America*	2535.3 (1645.7, 3628.2)		3599.4 (2315.0, 5160.8)	–84.07	7.25 (5.49, 10.57)	5.55 (4.25, 7.98)	–0.91 (–1.02, –0.81)
*Western Europe*	8314.2 (5941.7, 11 417.0)		6760.7 (4560.6, 9675.5)	–56.71	8.26 (6.67, 11.02)	5.98 (4.92, 8.38)	–1.09 (–1.21, –0.97)
*Western sub-Saharan Africa*	688.9 (340.7, 1156.4)		2227.0 (1199.1, 3621.5)	2667.79	2.10 (1.34, 3.75)	2.45 (1.73, 4.14)	0.55 (0.47, 0.63)

AAPC – average annual percent change, ASIR – age-standardised incidence rate, CI – confidence interval, GBD – Global Burden of Disease, SDI – sociodemographic index, UI – uncertainty interval

**Figure 1:**
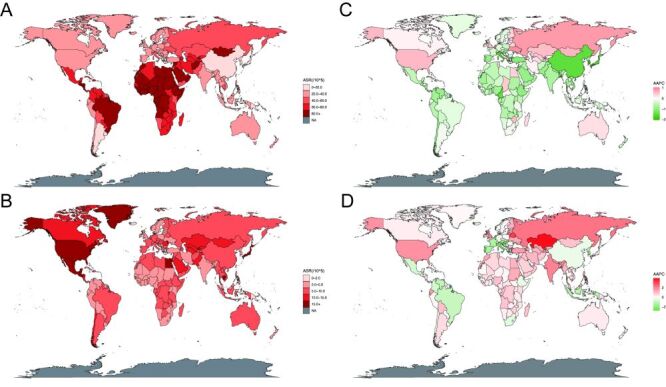
The ASIR and AAPC for AHC and HCV-related cirrhosis in 204 countries and territories. **Panel A.** The ASIR of AHC in 2019. **Panel B.** The ASIR of HCV-related cirrhosis in 2019. **Panel C.** The AAPC of ASIR in AHC from 1990–2019. **Panel D.** The AAPC of ASIR in HCV-related cirrhosis from 1990–2019. AAPC – average annual percent change, AHC – acute hepatitis C virus, ASIR – age-standardised incidence rate, HCV – hepatitis C virus

At the national level, the highest ASIR of AHC in women of reproductive age was recorded in Egypt (ASIR = 266.8; 95% UI = 215.84, 350.04), a country in sub-Saharan Africa. The lowest ASIR of AHC was observed in American Samoa (**Figure 1**, panel A, Table S1 in the **Online Supplementary Document**). In terms of HCV-related cirrhosis, the highest ASIR in women of reproductive age was observed in the USA (ASIR = 21.61; 95% UI = 17.79, 27.48). The lowest ASIR was observed in Papua New Guinea (**Figure 1**, panel B, Table S2 in the **Online Supplementary Document**). Overall, the ASIR of AHC and HCV-related cirrhosis in 2019 revealed significant regional and national disparities.

### The trend in the incidence of AHC and HCV-related cirrhosis among women of reproductive age from 1990–2019

Between 1990–2019, there was a significant increase in the global incidence cases of AHC (46.45%) and HCV-related cirrhosis (72.74%) among women of reproductive age. However, the global ASIR of AHC and HCV-related cirrhosis remained stable.

**Figure 2:**
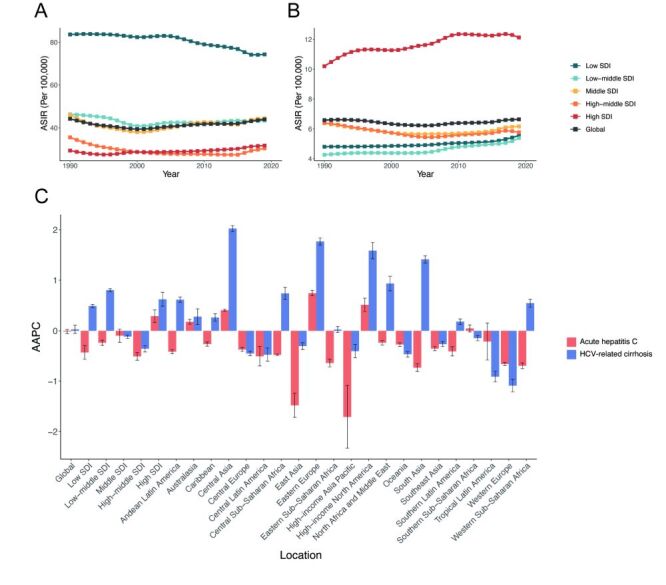
Temporal trends from 1990–2019 globally and among different regions. **Panel A.** Temporal trends in ASIR of AHC at global and SDI region levels. **Panel B.** Temporal trends in ASIR of HCV-related cirrhosis at global and SDI region levels. **Panel C.** AAPC of AHC and HCV-related cirrhosis at global, SDI, and GBD region levels. AAPC – average annual percent change, AHC – acute hepatitis C, ASIR – age-standardised incidence rate, GBD – Global Burden of Disease study, HCV – hepatitis C virus, SDI – sociodemographic index

In regions of different SDI categories, though the ASIR of AHC among reproductive-age women remained the highest in the low SDI region from 1990–2019, it displayed a favourable downward trend (AAPC = –0.43; 95% CI = –0.57, –0.29). The ASIR of AHC exhibited an increasing trend only in the high SDI regions (AAPC = 0.29; 95% CI = 0.16, 0.42) (**Figure 2**, panel A, **Table 1**). In contrast, the ASIR of HCV-related cirrhosis increased in the low, low-middle, and high SDI regions. Notably, the high SDI region exhibited the highest ASIR and demonstrated a significant upward trend during the study period (AAPC = 0.62; 95% CI = 0.49, 0.76) (**Figure 2**, panel B, **Table 2**). The marked increase in AHC and HCV-related cirrhosis in the high-SDI region is particularly concerning, signalling a worrying shift in the burden of disease to more developed areas.

Among the 21 GBD regions, only four regions saw a significant increase in ASIR of AHC among women of reproductive age, with the largest increase in Eastern Europe (AAPC = 0.75; 95% CI = 0.70, 0.80). Meanwhile, the greatest decrease in ASIR of AHC was found in the high-income Asia Pacific region. However, over half of the GBD regions showed an increasing trend in ASIR for HCV-related cirrhosis. The greatest increase was observed in Central Asia (AAPC = 2.02; 95% CI = 1.96, 2.08), followed by Eastern Europe and high-income North America. On the other hand, Western Europe witnessed the largest downward trend (**Figure 2**, panel C).

At the national level, Belgium experienced the greatest increase in the ASIR of AHC among women of reproductive age (AAPC = 1.04; 95% CI = 0.77, 1.31), followed by Ukraine and Russia. Conversely, the Republic of Korea witnessed the most substantial downward trend (**Figure 1**, panel C, Table S1 in the **Online Supplementary Document**). Regarding the trend of HCV-related cirrhosis, Kazakhstan underwent the largest increase in ASIR from 1990–2019 (AAPC = 3.97, 95% CI = 3.84, 4.09). The top five countries with the highest AAPC were located in Central Asia or Eastern Europe, calling for an urgent re-evaluation of health strategies in these regions. Meanwhile, the greatest downward trend was observed in Hungary (**Figure 1**, panel D, Table S2 in the **Online Supplementary Document**).

### Trends in the incidence of AHC and HCV-related cirrhosis across different age groups in women of reproductive age

Our analysis of temporal changes in AHC and HCV-related cirrhosis among specific age groups has unveiled noteworthy trends. Each age group contributed a relatively equivalent proportion to the overall incidence of AHC. From 1990–2019, a slight transition of incidence from younger to older populations was observed globally. (Figure S1 in the **Online Supplementary Document**). For HCV-related cirrhosis, individuals over 35 were the major contributors, with an increasing proportion of those aged 45–49 from 1990–2019, particularly in middle and high-middle regions (Figure S2 in the **Online Supplementary Document**).

**Figure 3:**
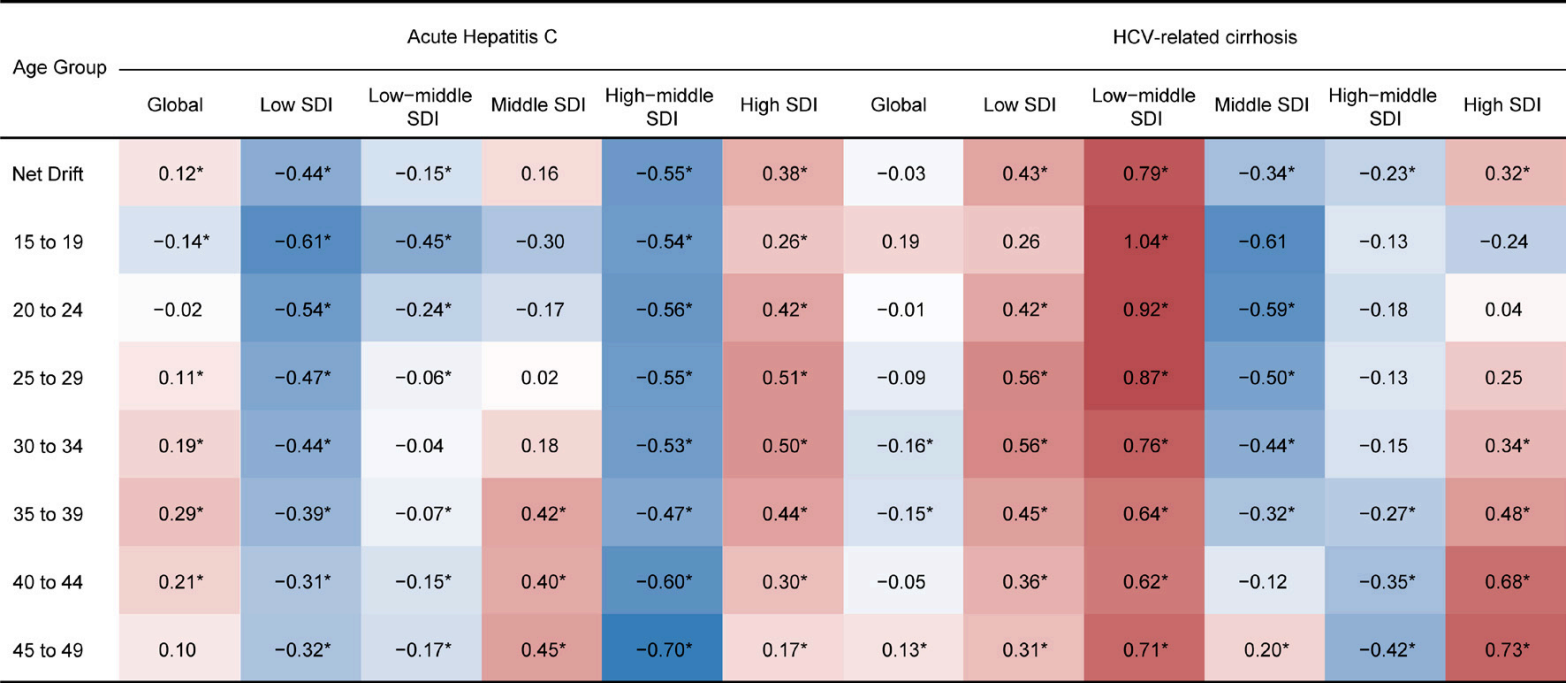
Local drifts with net drift for AHC and HCV-related cirrhosis globally and among different SDI regions. Red indicates a net drift or local drift above zero, while blue indicates a net or local drift below zero. The symbol ‘*’ refers to the result of the Wald test for the null hypothesis (‘Net drift = 0’/‘Local Drift = 0’) is not statistically significant, *P* < 0.05. AHC – acute hepatitis C, HCV – hepatitis C virus, SDI – sociodemographic index

The APC model revealed the annual percentage change in the age-specific incidence rate (local drift) of AHC and HCV-related cirrhosis. Globally, the largest increase in AHC incidence was observed in the 35–39 years group (local drift = 0.29; 95% CI = 0.17, 0.40). As to the five SDI regions, the incidence of AHC showed a favourable decreasing trend across all age groups in low, low-middle, and high-middle SDI regions. In terms of HCV-related cirrhosis, a significant increasing trend was only observed in the 45–49 years age group globally (local drift = 0.13; 95% CI = 0.04, 0.22). The five SDI regions showed varied patterns, with the incidence rate increasing across all age groups in low and low-middle regions and decreasing in most age groups in middle and high-middle regions. In the high SDI region, the local drift increased with age, reaching its peak in the 45–49 years group (**Figure 3**). The high incidence and increasing trend of HCV-related cirrhosis in the 45–49 years age group warrant further investigation into age-specific risk factors. Additionally, the divergent trends in the age-specific incidence rate across SDI regions indicate a complex interplay of various factors that require further investigation.

### Age, period, and cohort effects on the incidence of AHC and HCV-related cirrhosis among women of reproductive age

**Figure 4:**
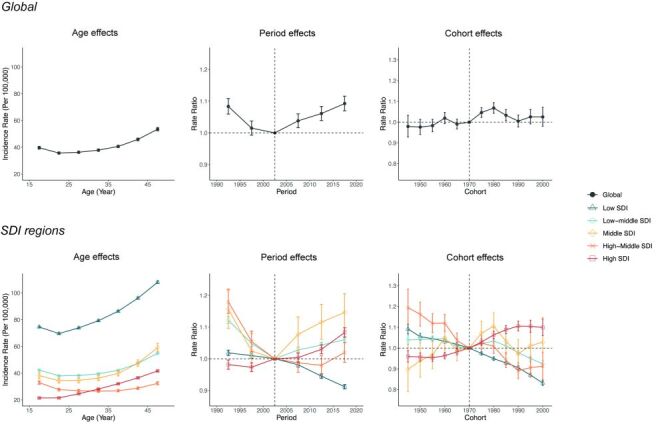
Parameter estimates of age, period, and cohort effects on AHC globally and among different SDI regions. Age effects are represented by fitted longitudinal age curves of AHC incidence (per 100 000 person-years), adjusted for period deviations. Period effects are demonstrated by the relative risk of AHC incidence for each period, compared to the reference period (2000–04), after adjusting for age and cohort effects. Cohort effects are shown by the relative risk of AHC incidence for each cohort, compared to the reference cohort (1970 cohort), after adjusting for age and period effects. AHC – acute hepatitis C, SDI – sociodemographic index

The APC model was used to estimate the impact of age, period, and cohort on the incidence of AHC and HCV-related cirrhosis among reproductive-age women globally and across five SDI regions. **Figure 4** shows the estimates of age, period, and cohort effects on AHC incidence. The age effects of AHC incidence were similar globally and in the low, low-middle, and middle SDI regions, declining from 15–19 years to 20–24 years and then rising. The relatively high incidence rate in the 15–19 age group is of particular concern, potentially indicating specific age-related risk factors for HCV infection.

The period effects on AHC incidence varied greatly across different SDI regions. Globally, the risk of AHC incidence declined from 1990–2004 but was followed by a continuous upward trend through 2019. Similar V-shaped trends appeared in low-middle, middle SDI regions, and high-middle SDI regions, but the high-middle SDI region did not experience the subsequent increase until 2014. The low SDI and high SDI regions exhibited completely opposite trends in the risk of AHC – consistently declining in the low SDI region, while increasing since 1994 in the high SDI region. The V-shaped trend in AHC incidence in most regions of the world, characterised by an initial decline and subsequent rise, signals the changing epidemiological dynamics over time.

As to cohort effects, the risk of AHC increased after the 1970 cohort but quickly decreased after the 1980 cohort globally. A resurgence in risk, however, was again observed for those born after 1990. Similar trends were observed in the middle SDI region. In contrast, the low and low-middle SDI regions saw a continued decline in AHC risk with younger birth cohorts, while the high SDI region showed consistent increases. The observed resurgence in AHC risk for those born after 1990 corresponds to the V-shaped trends seen in the period effect analysis, further underscoring the recurring tendency of AHC.

**Figure 5:**
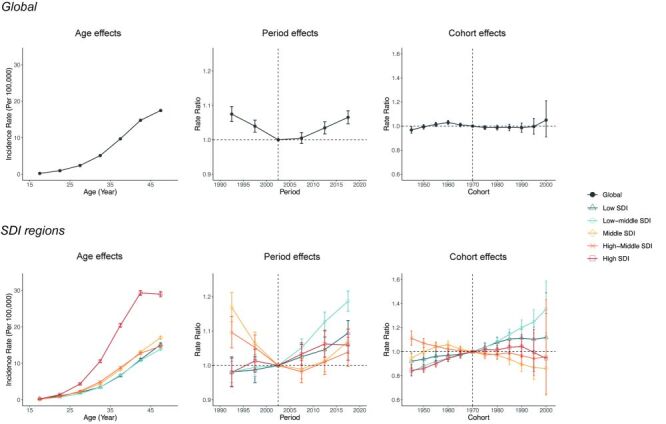
Parameter estimates of age, period, and cohort effects on HCV-related cirrhosis globally and among different SDI regions. Age effects are represented by fitted longitudinal age curves of HCV-related cirrhosis incidence (per 100 000 person-years), adjusted for period deviations. Period effects are demonstrated by the relative risk of HCV-related cirrhosis incidence for each period, compared to the reference period (2000–04), after adjusting for age and cohort effects. Cohort effects are shown by the relative risk of HCV-related cirrhosis incidence for each cohort, compared to the reference cohort (1970 cohort), after adjusting for age and period effects. HCV – hepatitis C virus, SDI – sociodemographic index

**Figure 5** displays the estimates of age, period, and cohort effects on the incidence of HCV-related cirrhosis. Globally, as well as in different SDI regions, the incidence rate of HCV-related cirrhosis generally rose with age. The period effects of HCV-related cirrhosis incidence mirrored those of AHC, with global risk exhibiting a V-shaped trend. Similar trends were observed in middle and high-middle regions. Meanwhile, the risk of HCV-related cirrhosis consistently rose in low and low-middle SDI regions. The high SDI region also observed a rising trend, but it plateaued from 2014–19. The persistent rise in HCV-related cirrhosis incidence in low and low-middle SDI regions, coupled with its resurgence globally, indicates an urgent need for targeted public health interventions.

In terms of cohort effects, the global risk for HCV-related cirrhosis remained stable for each successive younger birth cohort, with a slight increase for recent ones. However, the patterns varied across SDI regions. The low and low-middle regions witnessed increases in risk with each younger cohort, while middle and high-middle regions saw declines. The high SDI region exhibited an initial rise until the 1990 cohort, followed by a decrease that extended to the most recent cohort.

### Influential factors associated with ASIR and AAPC

Lastly, we examined factors potentially associated with the ASIR of AHC and HCV-related cirrhosis and their temporal trends from 1990–2019 among reproductive-age women. The ASIR of AHC in 2019 was negatively correlated with SDI (Pearson correlation coefficient (ρ) = –0.495; *P* < 0.001) (Figure S3, panel A in the **Online Supplementary Document**). This inverse relationship between ASIR of AHC and SDI is intriguing, as it suggests that higher development status may not be as protective against AHC. In contrast, no significant correlation existed between ASIR of HCV-related cirrhosis in 2019 and SDI (ρ = 0.080; *P* = 0.263) (Figure S3, panel B in the **Online Supplementary Document**).

In terms of the temporal trends in ASIR, a significant positive correlation between AAPC of AHC and SDI was found only in the countries with an SDI above 0.68, corresponding to high-middle and high SDI regions (ρ = 0.245; *P* = 0.017) (Figure S3, panel C in the **Online Supplementary Document**). The positive correlation further indicates that higher development status may be unexpectedly associated with rising AHC risk. On the other hand, a negative correlation was found between AAPC of HCV-related cirrhosis and SDI among all countries (ρ = –0.170; *P* = 0.016) (Figure S3, panel D in the **Online Supplementary Document**). The correlation between AAPC and ASIR in 1990 was also examined. A significant negative correlation was observed for both AHC (ρ = –0.329; *P* < 0.001) and HCV-related cirrhosis (ρ = –0.238; *P* < 0.001), suggesting that countries with higher initial incidences were more likely to experience a decreasing trend of incidence over time (Figure S4 in the **Online Supplementary Document**).

## DISCUSSION

In the study, we systematically analysed the spatial and temporal trends in the incidence of AHC and HCV-related cirrhosis among reproductive-age women from 1990–2019 at global, regional, and national levels. In addition, we employed an APC model to explore the effect of age, period, and cohort.

Globally, the incidence cases of AHC and HCV-related cirrhosis increased among reproductive-age women within the past 30 years, whereas the ASIR remained stable. Yet, the trend of ASIR varied considerably across SDI regions. Notably, the low, low-middle SDI region and the high SDI region displayed trends that merit attention and closer examination. Though the ASIR of AHC remained the highest in low SDI regions over the past 30 years, it displayed a promising downward trend. A similar decline was observed in the low-middle SDI region. The result was consistent with the negative correlation between ASIR in 1990 and AAPC for AHC, suggesting that the countries with higher initial AHC incidences were more likely to witness a decreasing trend over time. Several factors may have contributed to the decline of AHC incidence in reproductive-age women in regions with relatively low SDI. Since the WHO’s initiative to eliminate viral hepatitis by 2030, more than 120 countries have adopted a national viral hepatitis strategy, including many low SDI countries. Simplified guidelines, improved HCV testing and diagnostics, and reduced DAA treatment prices may be behind the decline [25]. Despite these advancements, it is important to underscore that the incidence rate of AHC among reproductive-age women in low SDI regions was still alarmingly high, almost double that of the global incidence rate. Sustained efforts to curb HCV infection are crucial in these areas, particularly with a focus on women, as they often face barriers to accessing health care services due to gender inequality. These barriers can manifest as restrictions on physical mobility, limiting women’s ability to reach clinics, as well as constraints on financial resources, which are often controlled by male relatives, hindering women’s autonomy in health care decisions. Furthermore, societal norms and expectations can impede women’s willingness and ability to prioritise their health needs and seek care [26]. On the other hand, the ASIR of HCV-related cirrhosis among reproductive-age women continued to rise in the low and low-middle SDI regions. Since the progression from HCV infection to cirrhosis generally takes 20–30 years, the trend may reflect the lagged impact of past infections [26]. Additionally, limited screening programs for liver diseases in chronic HCV patients due to inadequate health care resources, coupled with the still relatively high cost of the DAA regime, could further contribute to the increase in HCV-related cirrhosis [27].

Despite the abundance of health care capacity, high-SDI regions showed concerning trends in AHC and HCV-related cirrhosis incidence among reproductive-age women. The ASIR of AHC showed an upward trend, and more importantly, the ASIR of HCV-related cirrhosis continued to increase, starting from an already high initial value. Injection drug use remains the key risk factor for the emergence of HCV infections among reproductive-age women in high-SDI regions. Studies suggested that active injection drug use accounts for 1–42% of all HCV infections in high-income countries [28]. Moreover, female injection drug users (IDUs) are at a 1.2 times greater risk of HCV infection compared to their male counterparts [29], possibly due to their higher likelihood of engaging in high-risk injection behaviours, such as reliance on others for injection, borrowing used syringes, and having a regular IDU sex partner [30]. Therefore, gender-specific prevention strategies and harm-reduction measures could be key to reducing HCV transmission among reproductive-age women in high-SDI regions. In addition, the high incidence rate of HCV-related cirrhosis may be a lagged response to the high prevalence of chronic HCV infection. Though screening projects are relatively well-implemented in high SDI regions, the linkage to care and treatment after screening often proves inadequate, creating barriers to the early treatment of chronic HCV infection [31].

Patterns and trends of AHC and HCV-related cirrhosis incidence in reproductive-age women varied significantly across the 21 GBD regions and the 204 countries and territories. In 2019, the sub-Saharan Africa region reported the highest ASIR of AHC, with Egypt demonstrating the highest ASIR globally. Medical exposure has been identified as the major source of HCV transmission in Egypt, especially in women, since injecting drug use among women in this area is rare [32,33]. On a positive note, Egypt has been actively scaling its national DAA treatment program since 2014. Paired with HCV screening and other infection control strategies, the effort is expected to reduce new AHC cases and subsequent liver diseases [34,35].

As for HCV-related cirrhosis, high-income North America reported the highest ASIR in 2019 of all regions. This was probably driven by the USA, which recorded the highest ASIR among all nations. The high ASIR is likely due to a lagged impact of the 1945–65 birth cohort with significantly high HCV infection rates. It has been estimated that this specific birth cohort accounted for approximately 40% of the newly reported chronic HCV infections among United States (US) adults in 2018 [36]. Since 2012, the US Centres for Disease Control and Prevention has recommended HCV screening for individuals born during 1945–65 [37]. Expanded age-specific screening initiatives, coupled with the subsequent linkage-to-care system, have the potential to significantly reduce the incidence rate of HCV-related cirrhosis within this demographic.

Meanwhile, Eastern Europe and Central Asia, regions with close geographic proximity, experienced the largest increase in the ASIR of AHC and HCV-related cirrhosis among reproductive-age women, respectively. The increase was likely attributed to the large number of IDUs in these areas, with HCV prevalence among IDUs reaching as high as 90% in some regional cohorts [38]. Moreover, the coverage of harm-reduction programs – opioid agonist therapy and needle and syringe programs – is disappointingly low in these areas [39]. Russia, for example, completely bans the use of opioid agonist therapies and does not endorse needle and syringe programs [28,40]. The escalation of AHC and HCV-related cirrhosis incidence in Eastern Europe and Central Asia underscores the urgent need to broaden harm-reduction services. These services must be comprehensive, providing not only needle and syringe programs but also opioid agonist therapies where feasible, especially given the high prevalence of IDUs in these regions.

The incidence and trends of AHC and HCV-related cirrhosis among reproductive-age women showed considerable variation across different age groups. Globally, the incidence rate of AHC generally increased with age, except for the 15–19 age group, which reported a higher incidence rate than the subsequent 20–24 age group. The pattern was also pronounced in low, low-middle, and middle SDI regions, possibly due to the higher prevalence of young individuals engaging in active injection drug use in these regions. Supporting this, prior studies have suggested that countries with lower income tend to have younger IDU populations [41]. Furthermore, young IDUs often face an elevated risk of HCV acquisition, attributed to their low participation in harm-reduction programs and their propensity for high-risk injection behaviours [29]. These findings underscored the urgent need for age-appropriate prevention measures, especially in countries with lower SDI. For young IDUs, interventions should focus on increasing access to harm-reduction resources and education on safe injection practices to mitigate the risk of HCV transmission. By contrast, the incidence rate of HCV-related cirrhosis rose steadily with age among reproductive-age women. Attention is particularly warranted for the 45–49 age group, not only because this age group accounted for the largest proportion of cirrhosis incidence but also due to the significant increasing trend observed in most regions. According to the natural history of HCV infection, older cases are likely to progress to cirrhosis faster [31]. The insufficient screening programs and the lack of linkage-to-care efforts in many countries create further obstacles to the early treatment of HCV infection, rendering older HCV-infected populations more susceptible to cirrhosis [42]. Continued efforts in early detection and management of chronic HCV infection are essential to prevent progression to cirrhosis in older age groups.

The APC analysis offered further insights into the relative risks of AHC and HCV-related cirrhosis incidence among reproductive-age women across different time periods and birth cohorts. The relative risk of AHC exhibited a V-shaped trend, both globally and in varying SDI regions, indicating a recent resurgence in AHC incidence. The relative risk of AHC also showed a rebound in women born after 1990. With only 11 countries on track for WHO’s HCV elimination target by 2030 [31], the resurgence poses a significant global challenge. Concurrently, the relative risk of HCV-related cirrhosis either demonstrated a V-shape resurgence or a continued rise among different SDI regions. The rising risk in recent years could be attributed to the delayed impact of past infections combined with the emerging new infections resulting from the opioid crisis. This resurgence in AHC and HCV-related cirrhosis calls for a dynamic approach to HCV prevention and control, emphasising the continuous adaptation of strategies to the changing epidemiology of the disease.

To our knowledge, this study is the first to assess trends of AHC and HCV-related cirrhosis among reproductive-age women at global, regional, and national scales. While previous studies have explored the disease burden of hepatitis C using GBD 2019 data, our study uniquely focuses on the critical demographic of reproductive-age women.

Nevertheless, the present study acknowledges several limitations. First, our study shares the weakness of other GBD studies. In countries where data are absent or sparse, the estimates depend largely on the validity of the modelling efforts. The limitation in data might influence the accuracy of temporal trend estimation and APC analysis. Second, the data analysed were at a national level, limiting insights into subnational disease burden. Further research is needed to delve into more detailed subnational patterns. Third, while our analysis attempted to distinguish between period and cohort effects on incidence rates, the interpretation of APC results warrants caution due to the inherent collinearity between age, period, and cohort effects. Finally, the use of Pearson correlation might omit nonlinear relationships or systemic variations between variables. Future studies could consider employing time series analysis methods such as Autoregressive Integrated Moving Average models or Generalized Additive Models for further insights into nonlinear trends.

Given the evolving epidemiology of hepatitis C, recent guidelines have begun recommending HCV screening for all adults at least once in their lifetime and for all pregnant women during every pregnancy [43,44]. However, HCV screening programs are insufficient in many parts of the world, and subsequent linkage to care and treatment is inadequate. Gender inequality in many low-income countries frequently limits women’s access to health care. The escalating opioid crisis among reproductive-age women, especially younger females, exacerbates the situation. Our findings highlight the imperative for HCV prevention and care strategies tailored to the unique health challenges of reproductive-age women. A key component of this approach is the integration of HCV screening into standard women’s health services, such as gynaecological exams and prenatal care, to broaden testing coverage. Equally important is the adaptation of harm-reduction programs to offer gender-sensitive support and ensure women can access services in a stigma-free environment. Furthermore, establishing financial and social support systems, such as microfinance health loans and insurance schemes, could empower women, especially those with limited incomes, to independently seek and afford necessary health care. Educational campaigns to increase HCV knowledge among women and policy advocacy prioritising women’s health issues are also vital for promoting equitable health care access. In support of these strategies, we advocate for policy frameworks that facilitate international collaboration, leveraging successful interventions from various regions to enhance global HCV elimination efforts.

Future studies could aim to fill the epidemiological data gaps in regions with high or rising incidence rates and assess the effectiveness of current and potential new strategies for HCV prevention and treatment. It is also crucial to investigate the socio-behavioural factors that contribute to the spread of HCV among reproductive-age women and to evaluate the impact of educational campaigns on disease awareness.

## CONCLUSIONS

In conclusion, HCV infection in women of reproductive age presents significant public health threats, impacting both mothers and their infants during pregnancy. From 1990–2019, the incidence rate of AHC among reproductive-age women remained the highest in low SDI regions despite the observed downward trend. Unfavourable trends of HCV-related cirrhosis incidence were observed in low, low-middle, and high SDI regions, with high SDI regions recording the highest incidence rate throughout the study period. Attention should be given to the regions of sub-Saharan Africa, high-income North America, Eastern Europe, and Central Asia due to their high incidence rates or increasing trends of AHC and HCV-related cirrhosis. Females aged 15–19 years reported a higher incidence rate of AHC than the subsequent age group, and females aged 44–49 bore the highest burden of HCV-related cirrhosis across all age groups. Period effects suggested a resurgence in the risk of AHC and HCV-related cirrhosis in recent years, posing further challenges for HCV elimination.

## Additional material


Online Supplementary Document
