# Supplementary material for: Epidemiology of acute hepatitis C and hepatitis C virus-related cirrhosis in reproductive-age women, 1990–2019: An analysis of the Global Burden of Disease study
Source: J Glob Health. 2024 Apr 19;14:04077. doi: 10.7189/jogh.14.04077 (PMC11026988; doi:10.7189/jogh.14.04077)

Table S1. Incidence cases and age-standardized incidence rate of acute hepatitis C in women of reproductive age in 1990 and 2019, and its temporal trends, by countries or regions

Country or Region	Incidence Cases			Age-standardized Incidence Rate		
	Cases in 1990 (95% UI)	Cases in 2019 (95% UI)	Percentage Change	ASRs per 100,000 in 1990 (95% UI)	ASRs per 100,000 in 2019 (95% UI)	AAPC (95% CI)
Afghanistan	3234.1 (2372.5-4275.1)	9798.4 (7278.8-13143.9)	202.97%	138.61 (118.68-179.53)	121.11 (103.27-155.48)	-0.46 (-0.55 to -0.36)
Albania	201 (139.2-284.5)	126.1 (87.7-176.4)	-37.26%	24.42 (20.89-33.03)	19.67 (16.79-25.76)	-0.75 (-0.78 to -0.72)
Algeria	4712.2 (3537.1-6120.2)	8723.4 (6445.3-11563.1)	85.12%	89.92 (75.23-113.99)	76.77 (64.24-96.54)	-0.54 (-0.55 to -0.53)
American Samoa	1.2 (0.9-1.6)	1.4 (1-1.9)	16.67%	10.14 (8.47-12.94)	10.08 (8.42-13.05)	-0.03 (-0.16 to 0.11)
Andorra	4.6 (3.3-6.1)	7 (5-9.3)	52.17%	30.8 (25.77-39.84)	31.04 (25.87-39.83)	0.03 (0.02 to 0.04)
Angola	4061.3 (2950.7-5628.3)	10420.4 (7550.5-13824.3)	156.58%	190.77 (159.71-249.77)	158.5 (137.01-207.63)	-0.64 (-0.66 to -0.62)
Antigua and Barbuda	10.4 (7.5-14.1)	13 (9.3-17.1)	25%	64.82 (54.74-84.83)	52.33 (44.91-67.04)	-0.74 (-0.78 to -0.69)
Argentina	1404.4 (1031.5-1827.6)	2018.7 (1437.9-2674)	43.74%	17.58 (14.99-22.38)	16.96 (14.41-21.79)	-0.09 (-0.2 to 0.02)
Armenia	448.1 (310.9-618.5)	438 (310.4-612.7)	-2.25%	52.45 (44.41-68.49)	59.37 (50.61-79.51)	0.43 (0.4 to 0.47)
Australia	1625.1 (1149.5-2265.5)	2313.4 (1587.6-3184.7)	42.35%	36.29 (31.4-49.22)	38.84 (32.41-51.65)	0.24 (0.15 to 0.32)
Austria	613.4 (454.6-804.1)	571.1 (416.5-740.9)	-6.90%	30.5 (25.41-38.89)	27.17 (23.01-34.59)	-0.4 (-0.42 to -0.38)
Azerbaijan	1108.2 (779.5-1565.9)	1646.3 (1165-2249.8)	48.56%	59.48 (50.61-78.32)	60.59 (52.23-78.7)	0.1 (-0.08 to 0.27)
Bahamas	45.9 (33.6-61.5)	56 (41-74.9)	22%	64.18 (55.26-83.03)	52.91 (44.52-67.81)	-0.66 (-0.71 to -0.62)
Bahrain	96.5 (71.8-125)	270.1 (195.4-361.1)	179.90%	92.24 (78.8-116.99)	76.68 (64.92-97.71)	-0.63 (-0.66 to -0.59)
Bangladesh	5776.5 (4167.3-7780.3)	7362.6 (5383.2-9733.8)	27.46%	25.2 (21.65-32.51)	16.75 (14.14-21.73)	-1.41 (-1.5 to -1.32)
Barbados	36.4 (26.3-47.9)	34.7 (25-46.5)	-4.67%	53.46 (45.63-68.42)	47.86 (41.05-61.62)	-0.38 (-0.4 to -0.36)
Belarus	892.1 (615.5-1235.3)	831.9 (579.6-1184.1)	-6.75%	35.43 (29.67-46.36)	39.01 (33.33-52.55)	0.33 (0.25 to 0.41)
Belgium	544.1 (425.2-666.4)	789.8 (588.2-1031)	45.16%	22.02 (18.64-26.91)	29.97 (25.81-38.68)	1.04 (0.77 to 1.31)
Belize	30.9 (22.4-42.9)	72.2 (52.9-96.2)	133.66%	75.03 (64.5-100.65)	63.68 (54.23-82.08)	-0.57 (-0.6 to -0.53)
Benin	1415.9 (993.5-2007.3)	3420.1 (2443.2-4808.5)	141.55%	132.34 (113.87-174.14)	115.91 (101.25-155.61)	-0.46 (-0.5 to -0.42)
Bermuda	10 (7.1-13.7)	5.9 (4.2-8)	-41%	58 (50.47-75.94)	41.41 (35.32-53.31)	-1.15 (-1.18 to -1.12)
Bhutan	31.6 (22.9-41.6)	43.2 (30.9-58.1)	36.71%	24.18 (20.66-31.33)	21.76 (18.73-28.24)	-0.36 (-0.4 to -0.32)
Bolivia (Plurinational State of)	750.5 (543.2-1002.6)	1343 (954.6-1804.2)	78.95%	50 (42.42-64.76)	44.5 (36.53-57.7)	-0.4 (-0.41 to -0.39)
Bosnia and Herzegovina	273.2 (187.1-388.5)	176.9 (117.1-252.5)	-35.25%	23.34 (20.21-31.94)	23.24 (20.08-32.03)	-0.03 (-0.08 to 0.01)
Botswana	206.7 (151.7-276.9)	440 (321.3-596.2)	112.87%	68.13 (59.25-88.61)	66.96 (56.57-86.83)	-0.06 (-0.07 to -0.05)
Brazil	38417.2 (28056.6-50787.8)	56197.5 (40865-74348.1)	46.28%	99.58 (85.03-126.9)	95.73 (82.06-122.46)	-0.21 (-0.58 to 0.17)
Brunei Darussalam	13.2 (9.5-17.7)	24.7 (17.6-33)	87.12%	21.89 (18.37-28.26)	19.28 (15.96-25.77)	-0.44 (-0.46 to -0.42)
Bulgaria	424 (295.1-573.9)	297.6 (207.4-411.2)	-29.81%	20.33 (17.05-26.41)	20.05 (17.15-26.4)	-0.04 (-0.1 to 0.03)
Burkina Faso	2433.8 (1763.2-3444.2)	5067.4 (3703.7-6883)	108.21%	117.55 (99.26-155.69)	95.98 (80.79-123.66)	-0.76 (-0.82 to -0.69)
Burundi	1611.1 (1109.3-2295.9)	2582.3 (1832.1-3704.5)	60.28%	130.59 (112.1-174.61)	98.2 (81.45-131.1)	-0.97 (-1.01 to -0.93)
Cabo Verde	67.7 (49.3-92.1)	119.3 (89.4-157.3)	76.22%	88.33 (74.43-114.89)	82.31 (69.64-104.78)	-0.25 (-0.26 to -0.23)
Cambodia	1274.8 (808.1-1996.9)	2149 (1389.8-3230.2)	68.58%	47.85 (39.75-68.07)	48.38 (40.24-68.88)	0.04 (0.03 to 0.05)
Cameroon	3240.2 (2362.9-4270.4)	6241.8 (4641-8346)	92.64%	136.02 (115.11-176.19)	87.97 (74.06-113.3)	-1.52 (-1.61 to -1.43)
Canada	1847.2 (1329.1-2477.5)	2104.1 (1500.6-2798)	13.91%	24.78 (21.07-31.96)	25.3 (21.31-32.07)	0.1 (-0.02 to 0.23)
Central African Republic	897.1 (657.1-1195.3)	1865 (1362.8-2484.9)	107.89%	149.95 (124.53-190.25)	151.77 (127.26-195.63)	0.04 (0.01 to 0.06)
Chad	1380.7 (1005.9-1917.1)	4144.9 (2942.1-5717.1)	200.20%	106.81 (92.56-139.14)	119.8 (99.07-157.17)	0.4 (0.33 to 0.46)
Chile	978.2 (726.3-1268.9)	1019 (714-1344.8)	4.17%	28.14 (23.88-36.65)	21.36 (17.94-27.96)	-0.93 (-1.05 to -0.81)
China	73617.7 (48998-107177.4)	45604.5 (29472.6-68181.7)	-38.05%	21.76 (18.36-29.85)	13.78 (11.69-19.54)	-1.5 (-1.76 to -1.25)
Colombia	7142.2 (5307.2-9522.8)	8075.1 (6067.4-10913.5)	13.06%	81.23 (68.89-104.96)	64.09 (55.15-80.6)	-0.82 (-0.85 to -0.78)

Comoros	77.6 (57.4-104.6)	126.9 (91.9-171.1)	63.53%	78.31 (68.19-102.25)	70.29 (58.75-91.42)	-0.37 (-0.4 to -0.35)
Congo	880.7 (660.8-1160.4)	1995.8 (1473.4-2694.9)	126.62%	169.56 (141.2-219.39)	150.97 (127.89-196.94)	-0.44 (-0.54 to -0.34)
Cook Islands	0.7 (0.5-0.9)	0.5 (0.4-0.7)	-28.57%	14.23 (11.89-19.07)	12.36 (10.23-16.34)	-0.49 (-0.51 to -0.46)
Costa Rica	716.5 (550-945.5)	1090.9 (819-1460.2)	52.25%	91.77 (77.16-117.35)	85.03 (72.61-108.98)	-0.26 (-0.28 to -0.24)
Croatia	262.8 (187.7-354.3)	164.9 (114.5-231.2)	-37.25%	21.85 (18.34-28.45)	17.81 (15.19-23.38)	-0.7 (-0.74 to -0.65)
Cuba	1760.7 (1265.2-2313.8)	1258.5 (915-1677.9)	-28.52%	58.13 (49.24-74.3)	47.26 (40.63-61.13)	-0.71 (-0.75 to -0.66)
Cyprus	50.8 (36.7-66.2)	82.3 (57.5-108.8)	62.01%	25.5 (21.67-32.7)	22.22 (18.8-28.38)	-0.46 (-0.6 to -0.31)
Czechia	491.1 (358.2-677)	433.5 (304.5-582.1)	-11.73%	18.93 (15.98-24.9)	18.21 (15.27-23.66)	-0.15 (-0.2 to -0.09)
Cote d'Ivoire	2755.1 (2023.5-3673.1)	5686.3 (4209.8-7618.1)	106.39%	104.4 (89.12-137.11)	91.94 (79.38-118.99)	-0.44 (-0.46 to -0.42)
Democratic People's Republic of Korea	896.8 (591.4-1330.6)	886 (586.9-1287.7)	-1.20%	15.63 (13.15-21.91)	13.79 (11.88-18.79)	-0.43 (-0.45 to -0.41)
Democratic Republic of the Congo	13528.9 (9840.8-18423.1)	28603.9 (20964.8-38194.5)	111.43%	171.65 (146.55-226.22)	150.39 (127.07-192.45)	-0.46 (-0.47 to -0.44)
Denmark	362.9 (272.6-470.2)	339.7 (245.2-447.9)	-6.39%	27.03 (22.91-34.57)	25.72 (21.9-33.29)	-0.18 (-0.22 to -0.13)
Djibouti	74.2 (54.1-100.6)	198.8 (143.3-268.7)	167.92%	75.15 (62.68-97.16)	64.69 (54.42-83.71)	-0.52 (-0.55 to -0.49)
Dominica	11.5 (8.5-16.1)	8.3 (6.1-11)	-27.83%	68.38 (58.74-91.87)	49.96 (42.81-64.55)	-1.08 (-1.12 to -1.04)
Dominican Republic	1324.1 (977-1709.5)	1928.4 (1407.3-2631.5)	45.64%	72.39 (60.58-93.52)	67.83 (57.79-87.2)	-0.22 (-0.25 to -0.19)
Ecuador	975.9 (718.9-1287.7)	1768.2 (1295.3-2320.1)	81.19%	39.51 (33.24-50.52)	38.52 (32.13-49.56)	-0.08 (-0.12 to -0.05)
Egypt	28406 (19525-39503.6)	62288.9 (46069-80833.6)	119.28%	238.3 (197.72-328.92)	266.8 (215.84-350.04)	0.34 (0.17 to 0.5)
El Salvador	1301.4 (963.5-1712.8)	1497.2 (1114.3-1988.9)	15.05%	97.36 (81.12-126.92)	84.8 (71.89-108.21)	-0.48 (-0.55 to -0.4)
Equatorial Guinea	162.8 (118.6-221.2)	372.2 (277.7-486.5)	128.62%	176.11 (149.38-228.87)	120.42 (100.9-154.2)	-1.31 (-1.35 to -1.28)
Eritrea	561.7 (411.7-766.6)	1345.2 (995.4-1794.1)	139.49%	86.2 (75.49-113.47)	83.74 (72.05-111.23)	-0.1 (-0.11 to -0.08)
Estonia	133.8 (94.1-181.3)	100.4 (69.8-139.7)	-24.96%	35.41 (30.16-46.93)	35.84 (30.31-46.82)	0.05 (0 to 0.09)
Eswatini	124.9 (92.5-167)	209 (152.2-279.1)	67.33%	68.35 (59.97-90.15)	70.4 (60.12-92.05)	0.09 (0.07 to 0.12)
Ethiopia	11630.6 (8463.3-15385.2)	20112.1 (14815.6-26786.3)	72.92%	108.11 (91.87-138.41)	81.56 (70.97-106.11)	-0.98 (-1.13 to -0.83)
Fiji	34.8 (23.5-51.6)	39.7 (26.6-59.3)	14.08%	17.6 (14.66-24.07)	17.5 (15.02-24.07)	-0.02 (-0.08 to 0.04)
Finland	330.5 (245.5-431.1)	326.8 (240.1-432.5)	-1.12%	25.14 (21.22-31.75)	27.58 (23.62-35.71)	0.33 (0.27 to 0.38)
France	4155.3 (3136.8-5315.4)	3898.4 (2864.5-5241.3)	-6.18%	28.48 (24.66-36.66)	25.91 (21.87-33.52)	-0.33 (-0.39 to -0.27)
Gabon	384.3 (280.2-521.5)	712.8 (519.1-931.6)	85.48%	193.55 (162.24-248.68)	154.46 (129.01-198.43)	-0.78 (-0.9 to -0.67)
Gambia	198.5 (147.5-261.8)	495.2 (360.8-661.1)	149.47%	92.99 (78.38-119.3)	90.96 (77.23-119.64)	-0.09 (-0.14 to -0.04)
Georgia	735.8 (515.3-1019)	418.8 (296.8-581.4)	-43.08%	54.19 (46.15-71.91)	51.76 (43.78-67.5)	-0.13 (-0.29 to 0.03)
Germany	5331.8 (3970.2-6930.6)	4491.3 (3328.6-5845.7)	-15.76%	26.69 (22.58-33.63)	24.47 (20.77-31.15)	-0.27 (-0.65 to 0.1)
Ghana	4510.4 (3188.9-6250.6)	8605 (6136-11910)	90.78%	128.92 (108.9-166.35)	100.89 (86.73-131.08)	-0.85 (-0.95 to -0.76)
Greece	529.9 (383.5-682.7)	421.3 (308.7-562)	-20.49%	20.73 (17.79-26.17)	17.33 (14.76-22.09)	-0.62 (-1 to -0.23)
Greenland	6.1 (4.3-8.2)	4.9 (3.6-6.6)	-19.67%	40.75 (34.7-53.62)	38.14 (31.91-48.66)	-0.2 (-0.36 to -0.03)
Grenada	14.1 (10.2-19.3)	14.9 (11-19.9)	5.67%	73.47 (62.37-98.37)	56.89 (47.69-72.63)	-0.88 (-0.92 to -0.85)
Guam	4.3 (3-5.9)	4.2 (3-5.8)	-2.33%	12.45 (10.4-16.39)	10.88 (9.23-14.18)	-0.47 (-0.51 to -0.42)
Guatemala	2902.8 (2078-3987.2)	5931.8 (4344-7876.9)	104.35%	153.72 (131.02-205.93)	117.81 (101.71-151.69)	-0.91 (-0.96 to -0.86)
Guinea	1760.8 (1246.5-2439.3)	3490.8 (2511.8-4730.6)	98.25%	128.27 (113.07-172.53)	116.73 (100.04-152.17)	-0.34 (-0.38 to -0.29)
Guinea-Bissau	252.6 (186.2-343.6)	450.3 (331.6-609)	78.27%	109.29 (93.94-141.29)	93.18 (77.76-119.57)	-0.58 (-0.64 to -0.51)
Guyana	168 (119.5-230.2)	150.1 (109-203.9)	-10.65%	83.44 (70.56-109.85)	72.03 (62.58-95.69)	-0.51 (-0.56 to -0.47)
Haiti	1375.3 (964-1978.7)	2719.4 (1950.2-3711.6)	97.73%	89.48 (76.46-121.55)	79.63 (68.36-104.72)	-0.41 (-0.49 to -0.33)
Honduras	1639.3 (1156.3-2212)	3895 (2821.7-5122.4)	137.60%	147.64 (124.83-197.61)	140.39 (117.95-182.47)	-0.18 (-0.22 to -0.15)
Hungary	765.9 (509.6-1134.7)	494.4 (343.1-688.9)	-35.45%	30.49 (26.49-42.03)	22.38 (18.63-29.73)	-1.07 (-1.09 to -1.05)
Iceland	13.8 (10-18.1)	16.5 (12.3-21.5)	19.57%	21.51 (18.4-27.53)	20.29 (17.45-25.8)	-0.19 (-0.23 to -0.15)
India	49941.8 (34986.7-68604)	75654.2 (52141.8-103812)	51.48%	25.65 (21.78-33.82)	20.84 (17.52-27.7)	-0.71 (-0.92 to -0.5)
Indonesia	20611.9 (14465-28593.7)	27198.4 (19485-36567.9)	31.95%	41.46 (34.96-54.99)	38.81 (32.85-50.6)	-0.24 (-0.4 to -0.09)

Iran (Islamic Republic of)	7152.7 (5261.8-9380.6)	14473.7 (10488-19076.3)	102.35%	60.4 (51.93-77.29)	60.25 (51.13-76.21)	0.09 (-0.42 to 0.6)
Iraq	2864 (2180.6-3724.5)	7346.5 (5563.5-9484.9)	156.51%	82.44 (70.03-104.41)	68.81 (58.27-86.62)	-0.69 (-1.25 to -0.13)
Ireland	209.3 (152.5-279.5)	316.6 (227.5-411.1)	51.27%	23.88 (20.01-30.74)	25.33 (21.8-31.83)	0.21 (0.18 to 0.24)
Israel	300 (216.9-398)	581 (416.2-790.7)	93.67%	24.98 (20.84-32.15)	26.35 (22.47-34.37)	0.19 (0.17 to 0.21)
Italy	11215.4 (8335.8-14348.1)	6370.6 (4455.2-8465.1)	-43.20%	77.06 (65.85-97.25)	46.32 (39.75-59)	-1.71 (-1.8 to -1.62)
Jamaica	326.1 (232.7-428.6)	385.8 (282.6-523.4)	18.31%	55.27 (46.88-71.94)	50.01 (43.66-65.76)	-0.35 (-0.43 to -0.28)
Japan	8387.1 (5834.8-11213.2)	4513.6 (3117.5-6107.3)	-46.18%	23.82 (19.93-31.34)	15.1 (12.58-19.87)	-1.57 (-1.81 to -1.33)
Jordan	532.6 (401-683.1)	1571.3 (1209.7-2046.1)	195.02%	72.82 (61.91-91.57)	56.66 (48.37-71.54)	-0.89 (-1.02 to -0.77)
Kazakhstan	2092.7 (1494.6-2890.4)	2764.5 (1947.5-3902.9)	32.10%	51.23 (43.45-65.3)	59.37 (49.37-77.93)	0.51 (0.48 to 0.54)
Kenya	3270.7 (2369.8-4363.4)	7312.5 (5318.1-9636.6)	123.58%	67.88 (58.4-88.97)	58.12 (49.85-75.27)	-0.53 (-0.55 to -0.52)
Kiribati	4 (2.5-6)	6.6 (4.2-10.2)	65%	20.77 (17.55-29.61)	21.1 (18-29.7)	0.04 (-0.02 to 0.1)
Kuwait	251.4 (187.4-321.3)	817.7 (598.4-1080.4)	225.26%	63.23 (53.56-78.23)	56.21 (48.04-70.89)	-0.41 (-0.45 to -0.38)
Kyrgyzstan	669.4 (467.6-959.7)	1069.5 (748.4-1534.2)	59.77%	63.14 (54.1-85.49)	64.04 (54.84-86.04)	0.05 (0.04 to 0.07)
Lao People's Democratic Republic	431.9 (286.3-628.2)	783.7 (532.3-1125.3)	81.45%	41.82 (35.08-56.37)	39.67 (33.75-52.45)	-0.18 (-0.19 to -0.17)
Latvia	211.7 (152.4-286.5)	132.3 (95.2-180.5)	-37.51%	32.82 (28.12-42.4)	32.83 (28.24-42.7)	0.02 (-0.03 to 0.07)
Lebanon	448.7 (341.6-571.1)	671.2 (499.5-898.9)	49.59%	60.71 (51.69-74.05)	47.77 (41.22-60.29)	-0.9 (-1.01 to -0.8)
Lesotho	275.2 (204.2-376)	411.4 (295-545.1)	49.49%	68.76 (57.58-90.48)	75.84 (63.78-97.8)	0.35 (0.3 to 0.4)
Liberia	482.5 (352.1-660.9)	1193.7 (866.6-1605.8)	147.40%	113.29 (95.9-147.8)	98.09 (85.19-130.18)	-0.51 (-0.55 to -0.46)
Libya	1181.4 (840.8-1644.8)	2815.4 (1983-3853.1)	138.31%	152.03 (131.28-200.16)	138.56 (116.14-182.31)	-0.34 (-0.39 to -0.29)
Lithuania	349.6 (248.8-481.6)	240.1 (167.7-332.5)	-31.32%	38.14 (33.19-49.77)	40.45 (34.8-53.46)	0.24 (0.08 to 0.4)
Luxembourg	29.2 (21.6-39)	43.8 (32.1-57)	50%	29.2 (24.5-37.42)	27.35 (23.26-35.03)	-0.23 (-0.26 to -0.2)
Madagascar	1394 (1054.8-1815.8)	3830.7 (2855.8-5008.3)	174.80%	55 (47.39-69.96)	60.33 (51.52-76.33)	0.27 (-0.03 to 0.58)
Malawi	1899.1 (1374-2546.2)	3117.8 (2270.9-4161.7)	64.17%	91.08 (76.26-117.93)	71.67 (60.87-92.57)	-0.83 (-0.86 to -0.8)
Malaysia	1638.7 (1128.9-2381.2)	2907.2 (1998.5-4157.6)	77.41%	35.41 (30.21-47.43)	34.96 (30.24-46.83)	-0.05 (-0.1 to 0.01)
Maldives	17.5 (12.1-24.6)	29.9 (21-41.3)	70.86%	34.49 (29.46-45.17)	26.95 (22.87-35.26)	-0.86 (-0.89 to -0.83)
Mali	2515 (1800.4-3554.6)	5446.5 (3901.4-7573.1)	116.56%	133.58 (116.31-178.49)	113.49 (96.45-151.45)	-0.56 (-0.58 to -0.55)
Malta	24.3 (17.7-32.4)	23.4 (16.7-30.7)	-3.70%	24.9 (20.74-31.69)	23.27 (20.01-29.98)	-0.23 (-0.26 to -0.19)
Marshall Islands	1.8 (1.3-2.7)	2.5 (1.7-3.6)	38.89%	18.26 (15.49-24.89)	17.12 (14.46-22.93)	-0.23 (-0.26 to -0.2)
Mauritania	500.7 (365.2-673.2)	847.2 (619.2-1125.6)	69.20%	110.02 (93.09-144.61)	86.76 (73.34-110.15)	-0.82 (-0.83 to -0.8)
Mauritius	84.5 (60.3-118.8)	78.3 (57.3-104.7)	-7.34%	27.9 (23.35-36)	24.72 (20.28-32.1)	-0.43 (-0.5 to -0.36)
Mexico	18309.2 (13396.7-24453.5)	24599.3 (18040.9-32824.8)	34.35%	84.14 (72.43-109.38)	72.28 (62.5-93.02)	-0.53 (-0.75 to -0.3)
Micronesia (Federated States of)	4.1 (2.8-5.8)	4.3 (3-6)	4.88%	17.36 (14.49-23.31)	16.32 (14-21.73)	-0.21 (-0.23 to -0.2)
Monaco	1.9 (1.3-2.5)	2.4 (1.7-3.2)	26.32%	24.82 (20.77-31.74)	30.39 (25.69-38.13)	0.69 (0.65 to 0.74)
Mongolia	470.8 (306.4-728)	897.9 (568.6-1366.6)	90.72%	87.4 (73.61-125.55)	103.12 (89.59-149.8)	0.58 (0.5 to 0.67)
Montenegro	29.9 (20.7-41.7)	28.5 (20.1-39.5)	-4.68%	19.22 (16.18-25.5)	19.38 (15.94-25.18)	0.03 (0 to 0.06)
Morocco	6309.9 (4682.7-8378.6)	9584.7 (6997.2-12767.2)	51.90%	108.14 (92.73-139.84)	99.28 (86.17-129.08)	-0.32 (-0.51 to -0.12)
Mozambique	2000.1 (1474.8-2650.6)	4189.5 (3106.9-5640.3)	109.46%	67.95 (56.41-86.23)	62.88 (54.4-81.97)	-0.27 (-0.29 to -0.26)
Myanmar	3623.6 (2524.4-5117.9)	5003.8 (3526.4-7103.3)	38.09%	33.98 (28.74-44.74)	33.47 (29.1-44.01)	-0.05 (-0.06 to -0.04)
Namibia	200.7 (149.1-269.4)	364.8 (272.6-499.3)	81.76%	62.87 (53.89-81.85)	58.68 (49.79-75.72)	-0.24 (-0.27 to -0.22)
Nauru	0.4 (0.3-0.6)	0.5 (0.3-0.6)	25%	16.69 (14.23-22.68)	16.11 (13.79-21.75)	-0.12 (-0.14 to -0.11)
Nepal	837.7 (604.1-1113.2)	1366 (985.5-1825.1)	63.07%	19.47 (16.36-25.55)	16.05 (13.63-20.84)	-0.67 (-0.76 to -0.58)
Netherlands	592.2 (441.6-768.1)	590.7 (440.5-761)	-0.25%	14.69 (12.44-18.58)	14.99 (12.49-18.63)	0.06 (-0.27 to 0.38)
New Zealand	253.2 (174.9-342.6)	266.4 (185.4-357.9)	5.21%	28.06 (24.42-36.83)	25.11 (21.56-32.98)	-0.38 (-0.41 to -0.35)
Nicaragua	1258.8 (933.3-1606.3)	1774.7 (1319.5-2360.4)	40.98%	133.61 (112.18-171.01)	99.81 (85.8-130.43)	-0.99 (-1.18 to -0.79)
Niger	1962.6 (1412.5-2743.2)	4950.9 (3654.3-6892.5)	152.26%	115.46 (98.51-152.78)	105.02 (88.7-136.29)	-0.34 (-0.4 to -0.27)
Nigeria	25740.7 (18535.6-34697.1)	52922.1 (38634.4-70656.1)	105.60%	129.84 (110.11-167.65)	101.86 (86.39-131.46)	-0.82 (-0.9 to -0.75)

Niue	0.1 (0.1-0.1)	0.1 (0-0.1)	0%	15.38 (12.93-20.73)	14.42 (12.17-18.89)	-0.23 (-0.3 to -0.16)
North Macedonia	113.8 (78.5-159.2)	112.2 (77.5-157.1)	-1.41%	22.21 (19.05-29.43)	21.33 (18.14-28.23)	-0.14 (-0.16 to -0.12)
Northern Mariana Islands	2.4 (1.6-3.5)	1.4 (1-1.9)	-41.67%	17.26 (14.63-23.01)	14.15 (11.96-18.82)	-0.69 (-0.72 to -0.65)
Norway	260.7 (182.5-345.5)	292 (203-389.1)	12.01%	24.36 (20.79-32.12)	23.15 (19.56-30.32)	-0.14 (-0.25 to -0.04)
Oman	312 (234.9-408.2)	769.8 (562.1-999.8)	146.73%	101.14 (86.4-130.11)	81.82 (71.2-104.14)	-0.73 (-0.75 to -0.7)
Pakistan	14216.9 (10012.1-19998.3)	25657.6 (18025.6-35688.1)	80.47%	62.29 (53.75-84.11)	48.11 (41.56-65.97)	-0.9 (-1.3 to -0.49)
Palau	0.5 (0.4-0.7)	0.5 (0.3-0.7)	0%	12.94 (10.99-17.11)	12.49 (10.5-16.57)	-0.12 (-0.14 to -0.1)
Palestine	397.1 (298.6-521.5)	997.5 (745.6-1291.9)	151.20%	102.43 (86.16-130.72)	86.57 (75.06-110.5)	-0.58 (-0.59 to -0.57)
Panama	464.4 (344.6-606.2)	690.9 (517.7-894.3)	48.77%	75.18 (63.95-95.16)	66.36 (57.4-85.39)	-0.44 (-0.47 to -0.41)
Papua New Guinea	157.2 (103.3-232.1)	361.3 (240.2-538)	129.83%	15.87 (13.52-21.83)	14.43 (12.42-20.16)	-0.33 (-0.39 to -0.27)
Paraguay	780.4 (567.7-1058.9)	1411 (1048.7-1888.8)	80.80%	84.13 (70.64-108.28)	77.6 (65.94-98.25)	-0.26 (-0.35 to -0.18)
Peru	2313.7 (1665-3075.6)	3288.9 (2387.9-4383.8)	42.15%	43.54 (37.38-56.13)	36.8 (31.23-47.87)	-0.6 (-0.66 to -0.54)
Philippines	4438.4 (3102.6-6066.3)	7669 (5395.6-10452.4)	72.79%	28.21 (24.04-37.17)	26.68 (23.05-34.64)	-0.19 (-0.23 to -0.15)
Poland	2407.1 (1648.4-3365.7)	2017.2 (1363-2842.4)	-16.20%	25.76 (22.04-34.07)	22.65 (19.46-30.25)	-0.45 (-0.49 to -0.4)
Portugal	831.9 (604.5-1096.4)	662.7 (487.6-877)	-20.34%	32.72 (27.38-41.44)	25.54 (21.91-32.99)	-0.85 (-0.87 to -0.83)
Puerto Rico	626.5 (457.4-844.9)	420.6 (302.4-564.9)	-32.87%	65.41 (56.51-86.36)	50.94 (42.75-65.73)	-0.86 (-0.91 to -0.81)
Qatar	73.1 (54-97.6)	410.7 (301.2-549.8)	461.83%	96.85 (83.07-124.45)	84.68 (72.46-107.85)	-0.46 (-0.48 to -0.44)
Republic of Korea	3671.7 (2875.8-4547.1)	2337.4 (1661.2-3162.5)	-36.34%	30.89 (26.22-38.35)	16.6 (13.86-21.61)	-2.06 (-2.47 to -1.65)
Republic of Moldova	658 (454.6-949.2)	467.5 (310.3-660.8)	-28.95%	58.78 (50.86-80.26)	52.28 (44.48-70.2)	-0.4 (-0.43 to -0.36)
Romania	1613 (1093-2352.7)	1193.9 (801.7-1686.5)	-25.98%	28.68 (24.64-39.32)	28.08 (23.83-38.02)	-0.06 (-0.17 to 0.06)
Russian Federation	13663.6 (9528.9-18843.5)	15706.7 (11033.1-21623.6)	14.95%	37.29 (31.75-48.99)	46.74 (39.87-60.86)	0.8 (0.75 to 0.84)
Rwanda	1676.7 (1218.8-2307.1)	2489.7 (1847.1-3339)	48.49%	108.24 (93.48-140.98)	78.66 (68.44-103.72)	-1.1 (-1.13 to -1.06)
Saint Kitts and Nevis	7.7 (5.5-10.4)	8.7 (6.3-11.7)	12.99%	78.61 (65.8-104.46)	55.2 (47.7-71.94)	-1.22 (-1.27 to -1.17)
Saint Lucia	25.2 (18.1-33.9)	27.6 (19.6-36.6)	9.52%	75.32 (64.39-99.4)	58.36 (51.05-77.15)	-0.89 (-0.93 to -0.84)
Saint Vincent and the Grenadines	16.7 (12.1-22.5)	15 (10.7-19.9)	-10.18%	64.49 (55.22-84.88)	53.12 (44.99-67.99)	-0.67 (-0.7 to -0.64)
Samoa	3.9 (2.7-5.3)	5.1 (3.7-7)	30.77%	10.67 (8.86-14.16)	10.22 (8.46-13.17)	-0.18 (-0.32 to -0.04)
San Marino	1.5 (1.1-2)	2.3 (1.7-3)	53.33%	25.98 (21.91-33.29)	26.72 (22.21-33.39)	0.1 (0.06 to 0.13)
Sao Tome and Principe	25 (18.4-33.7)	43.3 (32.4-58.1)	73.20%	101.32 (87.11-131.37)	83.56 (72.04-107.85)	-0.66 (-0.68 to -0.64)
Saudi Arabia	5210 (3711.6-7205.5)	14025.2 (9894.4-19360)	169.20%	181.08 (152.26-243.34)	140.36 (119.26-183.76)	-0.91 (-1.11 to -0.71)
Senegal	1077.8 (836.2-1352.5)	2417.3 (1821.6-3107.8)	124.28%	65.37 (55.97-81.61)	67.29 (57.52-86.65)	0.08 (0.02 to 0.15)
Serbia	507.6 (346-711.8)	421.5 (296.3-582.3)	-16.96%	22.36 (18.89-29.56)	21.35 (18.12-28.15)	-0.15 (-0.17 to -0.14)
Seychelles	5.8 (4.1-7.9)	7.1 (5.1-9.8)	22.41%	30.85 (27.09-40.27)	29.67 (24.89-38.92)	-0.13 (-0.15 to -0.11)
Sierra Leone	903.4 (648.3-1246.7)	1939.8 (1408.4-2641.4)	114.72%	106.78 (90.95-139.59)	94.77 (78.25-121.4)	-0.41 (-0.46 to -0.37)
Singapore	165.6 (114.1-229.1)	255.3 (172-344.4)	54.17%	17.96 (15.46-23.74)	15.75 (13.25-20.56)	-0.44 (-0.47 to -0.41)
Slovakia	321.2 (220.9-450.4)	288.5 (199.3-404.8)	-10.18%	24.29 (20.86-32.39)	22.39 (19.19-29.99)	-0.28 (-0.34 to -0.21)
Slovenia	130.8 (91-186.6)	93.9 (65.7-132.9)	-28.21%	26.46 (22.45-35.06)	21.65 (18.18-28.45)	-0.7 (-0.74 to -0.66)
Solomon Islands	9 (6.6-12)	21.9 (15.8-29.7)	143.33%	11.66 (9.81-15.01)	13.16 (10.94-17.28)	0.4 (0.28 to 0.51)
Somalia	1426.2 (1036.1-1903.4)	3920.7 (2836.3-5374.6)	174.91%	95.18 (79.66-123.99)	90.17 (75.25-119.59)	-0.2 (-0.28 to -0.13)
South Africa	5883.6 (4258-7822.4)	9173.9 (6567.2-12237.3)	55.92%	62.4 (53.55-80.85)	60.89 (50.7-80.22)	-0.1 (-0.2 to 0)
South Sudan	928.2 (686.2-1243)	1449.8 (1057.5-1920.8)	56.19%	77.13 (66.52-99.25)	68.94 (60.24-88.83)	-0.38 (-0.4 to -0.36)
Spain	3374.9 (2531.4-4313.8)	3140.4 (2293-4257.8)	-6.95%	35.1 (28.9-45.14)	27.5 (23.41-35.91)	-0.9 (-1.07 to -0.74)
Sri Lanka	942.2 (687-1254.1)	966.3 (709.2-1278.1)	2.56%	20.35 (17.05-25.61)	17.37 (14.78-22.17)	-0.57 (-0.82 to -0.33)
Sudan	4852.3 (3600.4-6516.3)	9203 (6768.9-12120.8)	89.66%	113.98 (96.87-148.26)	92.85 (78.66-117.36)	-0.7 (-0.72 to -0.69)
Suriname	68.3 (50.1-91.1)	86.6 (62.9-116.8)	26.79%	71.41 (60.3-92.04)	59.31 (51.44-78.73)	-0.64 (-0.67 to -0.61)
Sweden	546.3 (383.9-721.5)	540.5 (374.3-728.8)	-1.06%	25.58 (21.58-33.57)	23.72 (20.14-31.04)	-0.25 (-0.34 to -0.15)
Switzerland	424.6 (311.8-556.8)	529.7 (389-681)	24.75%	23.34 (20-29.9)	25.45 (21.53-32.39)	0.31 (0.25 to 0.38)

Syrian Arab Republic	3085.9 (2258.3-4108.4)	4512.6 (3244-6050.5)	46.23%	125.64 (106.29-163.76)	114.6 (99.68-149.06)	-0.3 (-0.37 to -0.22)
Taiwan (Province of China)	944.2 (612.8-1411.9)	750.3 (482.4-1135.5)	-20.54%	17.23 (14.48-24.2)	13.96 (11.72-19.25)	-0.75 (-0.83 to -0.66)
Tajikistan	794.4 (543.3-1173)	1681.7 (1180.6-2375.7)	111.69%	63.44 (54.37-85.65)	68.58 (58-91.25)	0.28 (0.26 to 0.3)
Thailand	5443.3 (3853.4-7533)	4641.8 (3351.1-6297.2)	-14.72%	33.53 (28.31-44.08)	26.1 (22.57-34.18)	-0.85 (-0.94 to -0.75)
Timor-Leste	70.7 (47.6-99.8)	115.4 (81.9-158.6)	63.22%	36.89 (30.69-49.75)	33.71 (28.74-44.37)	-0.32 (-0.34 to -0.29)
Togo	886.3 (639-1203.5)	1808.6 (1324.2-2409.2)	104.06%	107.16 (89.82-139.68)	90.08 (77.42-117.47)	-0.6 (-0.63 to -0.56)
Tokelau	0.1 (0-0.1)	0 (0-0.1)	-100%	17.04 (14.36-22.68)	14.51 (12.17-19.27)	-0.56 (-0.6 to -0.52)
Tonga	4.4 (2.9-6.4)	4.7 (3.2-6.6)	6.82%	19.33 (16.37-26.29)	18.68 (15.69-24.84)	-0.12 (-0.14 to -0.1)
Trinidad and Tobago	202.8 (147.7-272)	173.2 (125.6-233.9)	-14.60%	66.56 (56.31-87.06)	50.21 (42.37-64.2)	-0.97 (-1 to -0.95)
Tunisia	877.1 (688.8-1106.6)	1634.4 (1215.3-2172.6)	86.34%	46.71 (40.79-57.86)	51 (44.02-65.81)	0.31 (0.2 to 0.41)
Turkey	7284.1 (5524.6-9380.6)	8882.7 (6829.3-11407.5)	21.95%	53.04 (44.47-65.91)	39.05 (33.04-48.8)	-1.07 (-1.23 to -0.92)
Turkmenistan	570.2 (390.8-815.6)	834.2 (579.5-1176.2)	46.30%	63.32 (52.81-84)	67.75 (57.54-89.71)	0.24 (0.21 to 0.27)
Tuvalu	0.4 (0.3-0.6)	0.4 (0.3-0.6)	0%	17.39 (14.6-23.53)	15.46 (13.2-20.46)	-0.41 (-0.43 to -0.38)
Uganda	3302.8 (2338.1-4568.3)	6934.5 (5142.9-9345.3)	109.96%	91.56 (77.58-120.77)	75.96 (65.27-98.05)	-0.65 (-0.67 to -0.62)
Ukraine	4834.4 (3393-6638.7)	4997.7 (3474.6-7128)	3.38%	38.46 (33.23-50.36)	49.6 (43.13-66.52)	0.88 (0.85 to 0.92)
United Arab Emirates	261.2 (192.7-342.3)	1434.6 (1013.9-1945.9)	449.23%	82.81 (71.52-105.51)	75.62 (64.54-96.64)	-0.32 (-0.34 to -0.3)
United Kingdom	3817.2 (2708.3-5081.6)	4613.7 (3268.4-6106.4)	20.87%	26.4 (22.67-34.52)	29.26 (24.76-37.81)	0.39 (0.1 to 0.68)
United Republic of Tanzania	3941.4 (2919.8-5296.4)	9147.6 (6748.3-12340.2)	132.09%	70.47 (59.63-88.86)	68.39 (58.34-89.59)	-0.1 (-0.12 to -0.09)
United States of America	19818.4 (13962.4-26648.5)	25821.8 (18716.5-34153.3)	30.29%	29.5 (25.16-38.41)	33.94 (28.81-43.98)	0.54 (0.41 to 0.68)
United States Virgin Islands	18.6 (13.3-24.8)	12.5 (8.9-16.4)	-32.80%	63.88 (54.87-81.41)	53.97 (46.19-70.09)	-0.58 (-0.59 to -0.57)
Uruguay	121.3 (88-161.6)	128.6 (90.6-172.6)	6.02%	16.12 (13.61-21.07)	14.98 (12.35-19.15)	-0.25 (-0.27 to -0.23)
Uzbekistan	3335.4 (2260-4779.8)	6578.5 (4379.7-9471.2)	97.23%	66.52 (56.29-89.67)	73.48 (62.2-99.72)	0.35 (0.33 to 0.37)
Vanuatu	6.2 (4.3-8.8)	12.2 (8.4-17.2)	96.77%	17.43 (14.94-23.74)	16.46 (13.71-21.68)	-0.21 (-0.29 to -0.14)
Venezuela (Bolivarian Republic of)	4183 (3160.9-5569)	4880.7 (3607.4-6597.2)	16.68%	85.68 (73.6-110.9)	66.63 (56.75-86.17)	-0.89 (-1.03 to -0.75)
Viet Nam	7244.1 (5017.3-10438.1)	7820.5 (5480.6-10809.2)	7.96%	40.2 (33.79-54.19)	30.36 (25.34-39.81)	-0.94 (-1.03 to -0.85)
Yemen	1730.3 (1319.2-2209)	4184.2 (3204.4-5412.3)	141.82%	70.61 (60.62-87.07)	57.88 (49.38-71.93)	-0.73 (-1.28 to -0.18)
Zambia	1592.6 (1167.2-2165)	3314.5 (2480.3-4569.9)	108.12%	92.21 (75.68-120.37)	76.47 (63.85-99.07)	-0.65 (-0.69 to -0.6)
Zimbabwe	1462.2 (1059.6-1982.5)	2879 (2051.6-4065.5)	96.90%	67.02 (55.8-88.35)	77.7 (66.4-103.98)	0.52 (0.43 to 0.6)

Abbreviations: ASR, age-standardized rate; AAPC, average annual percent change; UI, uncertainty interval; CI, confidence interval.

Table S2. Incidence cases and age-standardized incidence rate of cirrhosis due to hepatitis C in women of reproductive age in 1990 and 2019, and its temporal trends, by countries or regions

Country or Region	Incidence Cases			Age-standardized Incidence Rate		
	Cases in 1990 (95% UI)	Cases in 2019 (95% UI)	Percentage Change	ASRs per 100,000 in 1990 (95% UI)	ASRs per 100,000 in 2019 (95% UI)	AAPC (95% CI)
Afghanistan	108.2 (51.5-178.7)	449.1 (245.5-689.3)	315.06%	5.22 (3.32-8.88)	6.64 (4.61-10.84)	0.84 (0.54 to 1.15)
Albania	34.7 (20.9-52.5)	36.4 (21.8-56.7)	4.90%	5.3 (4.02-8.01)	5.28 (3.83-8.04)	0.04 (-0.09 to 0.17)
Algeria	195.6 (116.6-295.7)	651.2 (396-943.8)	232.92%	4.77 (3.42-7.49)	5.62 (4.06-8.24)	0.57 (0.44 to 0.69)
American Samoa	0.2 (0.1-0.4)	0.3 (0.2-0.5)	50%	2.46 (1.82-3.92)	2.58 (1.99-4.09)	0.12 (0.04 to 0.21)
Andorra	1 (0.6-1.5)	1.5 (0.9-2.4)	50%	6.29 (4.79-9.57)	5.93 (4.79-9.01)	-0.21 (-0.23 to -0.19)
Angola	107.1 (54.7-176.7)	430.7 (255.9-662.5)	302.15%	5.79 (3.72-10.04)	7.31 (5.05-11.46)	0.86 (0.74 to 0.98)
Antigua and Barbuda	0.5 (0.3-0.8)	1 (0.6-1.6)	100%	3.54 (2.66-5.47)	3.57 (2.7-5.59)	0.05 (-0.16 to 0.27)
Argentina	472 (311.8-668.2)	836.6 (547.5-1207.7)	77.25%	5.97 (4.7-8.63)	6.82 (5.27-9.85)	0.49 (0.38 to 0.6)
Armenia	17.4 (10.1-28.2)	34.6 (20-54.6)	6371.26%	2.35 (1.79-3.77)	4.16 (3.14-6.55)	2 (1.91 to 2.09)
Australia	235.6 (166.3-315.7)	361.2 (254-482.6)	53.31%	5.1 (4.01-7.07)	5.57 (4.42-7.71)	0.28 (0.15 to 0.41)
Austria	184.6 (111.6-276.6)	159.8 (97.9-243.1)	-13.43%	9.11 (7.27-13.29)	6.84 (5.52-10.33)	-0.99 (-1.06 to -0.91)
Azerbaijan	76.3 (44.5-120.4)	192.7 (105.8-316.2)	152.56%	5.46 (4.02-8.78)	6.61 (4.71-10.83)	0.68 (0.6 to 0.76)
Bahamas	2.9 (1.7-4.4)	4.6 (2.6-7.1)	4903.45%	4.66 (3.56-7.12)	4.04 (3.05-6.18)	-0.49 (-0.55 to -0.44)
Bahrain	4.9 (2.9-7.2)	26.2 (15.6-38.1)	434.69%	6.09 (4.3-9.52)	6.77 (5.03-9.92)	0.37 (0.31 to 0.42)
Bangladesh	503.6 (279.3-841.8)	1607.9 (903.2-2707.7)	219.28%	2.86 (1.98-4.81)	3.84 (2.78-6.31)	1.04 (1 to 1.09)
Barbados	1.6 (0.9-2.5)	1.9 (1.1-3)	18.75%	2.45 (1.82-3.93)	2.3 (1.8-3.47)	-0.22 (-0.29 to -0.16)
Belarus	57.4 (33.2-90.7)	141.2 (82.1-218.6)	145.99%	2.19 (1.71-3.45)	5.32 (4.19-8.18)	3.13 (2.96 to 3.29)
Belgium	243 (158.3-348.2)	259.8 (160-391.5)	6.91%	9.37 (7.45-13.65)	9 (7.04-13.3)	-0.15 (-0.23 to -0.07)
Belize	1.4 (0.8-2.1)	5.7 (3.3-8.9)	307.14%	4.85 (3.57-7.54)	5.62 (4.16-8.69)	0.49 (0.42 to 0.55)
Benin	19.5 (9.2-34.2)	60.3 (33.1-100.4)	209.23%	2.34 (1.53-4.38)	2.48 (1.78-4.34)	0.2 (0.12 to 0.28)
Bermuda	0.5 (0.3-0.8)	0.3 (0.2-0.5)	-40%	2.82 (2.08-4.34)	1.98 (1.56-3.13)	-1.19 (-1.33 to -1.04)
Bhutan	3.2 (1.7-5.4)	8.3 (4.6-13.5)	159.38%	3.21 (2.24-5.73)	4.47 (3.25-7.26)	1.17 (1.11 to 1.24)
Bolivia (Plurinational State of)	29.6 (15-52)	87.8 (46.3-152.1)	196.62%	2.43 (1.71-4.19)	3.14 (2.22-5.3)	0.92 (0.8 to 1.05)
Bosnia and Herzegovina	61.4 (36.9-94.5)	39 (23.6-61.6)	-92.67%	5.48 (4-8.27)	4.28 (3.29-6.34)	-0.81 (-0.99 to -0.64)
Botswana	12.9 (7.5-20.9)	34.1 (19.8-53.8)	49.61%	4.85 (3.46-7.95)	5.18 (3.98-8.32)	0.28 (0.04 to 0.52)
Brazil	2506.2 (1618.3-3595.2)	3537.2 (2261.2-5082)	41.14%	7.34 (5.51-10.76)	5.6 (4.18-8.2)	-0.92 (-1.03 to -0.81)
Brunei Darussalam	3.7 (2.3-5.8)	7.4 (4.6-11.2)	100%	6.95 (5.28-10.3)	5.66 (4.34-8.57)	-0.71 (-0.91 to -0.52)
Bulgaria	99.8 (59-154.1)	103.7 (64.7-159.1)	-65.33%	4.25 (3.35-6.42)	5.44 (4.33-8.17)	0.86 (0.81 to 0.91)
Burkina Faso	31.2 (15.7-54.2)	95.1 (46.7-171.2)	204.81%	1.84 (1.29-3.39)	2.13 (1.47-3.85)	0.63 (0.33 to 0.93)
Burundi	45.5 (25.2-73.1)	110.4 (63-174.1)	142.64%	4.57 (3.13-7.76)	5.29 (3.75-8.64)	0.51 (0.45 to 0.58)
Cabo Verde	1 (0.5-1.7)	2.7 (1.6-4.6)	170%	1.71 (1.16-3.09)	2 (1.58-3.4)	0.55 (0.49 to 0.61)
Cambodia	306.1 (180.4-476.3)	714.5 (426.7-1081)	133.42%	14.97 (10.56-24.2)	17.32 (12.66-26.36)	0.48 (0.34 to 0.61)
Cameroon	37.7 (18.8-65.2)	128.2 (66.5-220.9)	240.05%	2.05 (1.42-3.72)	2.18 (1.53-3.83)	0.22 (0.12 to 0.33)
Canada	995.5 (692.1-1324.9)	1277.7 (906.3-1715.2)	28.35%	12.63 (9.9-16.93)	13.54 (10.78-18.11)	0.24 (0.22 to 0.26)
Central African Republic	33 (18.6-52.5)	76.7 (45.6-119.5)	132.42%	6.28 (4.34-11.04)	6.97 (4.73-11.46)	0.36 (0.31 to 0.4)
Chad	27.1 (13.1-48.4)	72.2 (38.5-121.5)	166.42%	2.61 (1.59-4.9)	2.69 (1.8-4.63)	0.12 (0.03 to 0.2)
Chile	430.2 (270.3-626.3)	642.3 (414.3-941.4)	49.30%	13.41 (10.04-19.92)	12.62 (9.3-18.15)	-0.23 (-0.29 to -0.16)
China	17138.5 (10534.4-25532.3)	25267.9 (16509-36200.1)	47.43%	6.32 (4.51-9.5)	5.79 (4.33-8.22)	-0.29 (-0.37 to -0.21)
Colombia	397.3 (257.7-580.2)	609.1 (374-919.6)	53.31%	5.83 (4.28-8.63)	4.73 (3.73-7.02)	-0.68 (-0.86 to -0.5)

Comoros	4.5 (2.6-7.4)	10.6 (6-16.7)	135.56%	5.53 (3.76-9.35)	6.3 (4.69-9.98)	0.45 (0.42 to 0.47)
Congo	22.8 (12.5-36.6)	82.4 (48.5-127.4)	261.40%	5.08 (3.51-8.69)	6.51 (4.65-10.42)	0.92 (0.8 to 1.04)
Cook Islands	0 (0-0.1)	0.1 (0-0.1)	Inf%	1.13 (0.88-1.82)	1.3 (0.96-2.05)	0.5 (0.44 to 0.55)
Costa Rica	64.3 (42.3-92.5)	130.5 (82.1-187.3)	102.95%	10.48 (8.02-15.26)	9.89 (7.41-14.59)	-0.2 (-0.21 to -0.18)
Croatia	122.9 (75.1-185.1)	65.3 (40.4-97.3)	-46.87%	9.35 (6.98-13.97)	5.83 (4.51-8.67)	-1.64 (-1.71 to -1.57)
Cuba	85.7 (50.6-132.2)	103.2 (59.9-160.6)	20.42%	3.1 (2.4-4.91)	3.25 (2.45-5.01)	0.18 (0.13 to 0.23)
Cyprus	10.1 (6-16)	18.6 (11.2-29.7)	84.16%	4.96 (3.83-7.73)	4.43 (3.42-6.83)	-0.41 (-0.5 to -0.33)
Czechia	144.4 (91.1-214.5)	172.1 (104.6-258.5)	19.18%	4.94 (3.83-7.32)	5.6 (4.39-8.17)	0.43 (0.28 to 0.58)
Cote d'Ivoire	41.8 (20.9-71.1)	120.6 (63.8-205.5)	188.52%	2.08 (1.3-3.87)	2.27 (1.61-4.05)	0.31 (0.25 to 0.37)
Democratic People's Republic of Korea	285 (165.8-449.4)	338.4 (188.2-544.6)	428.95%	5.5 (3.99-8.5)	4.53 (3.33-7.02)	-0.66 (-0.74 to -0.58)
Democratic Republic of the Congo	417.9 (235.4-653.3)	1249.3 (771.1-1853.7)	198.95%	6.08 (4.23-10.06)	7.46 (5.63-11.72)	0.74 (0.63 to 0.86)
Denmark	64.7 (39.4-98)	79.5 (48-120.3)	22.87%	4.73 (3.73-7.11)	5.71 (4.64-8.72)	0.67 (0.51 to 0.82)
Djibouti	4.8 (2.8-7.7)	18.1 (10.5-29.2)	277.08%	6.18 (4.39-10.21)	6 (4.39-9.53)	-0.02 (-0.12 to 0.09)
Dominica	0.5 (0.3-0.8)	0.5 (0.3-0.8)	0%	3.84 (2.89-6.15)	3.14 (2.41-4.87)	-0.67 (-0.84 to -0.5)
Dominican Republic	77.9 (44.9-121.2)	172.2 (97-268.1)	121.05%	5.59 (4.18-8.96)	6.33 (4.81-9.89)	0.45 (0.3 to 0.59)
Ecuador	42.4 (24.5-69.7)	147.2 (83-232.6)	247.17%	2.17 (1.62-3.47)	3.37 (2.42-5.28)	1.51 (1.41 to 1.61)
Egypt	1441.1 (883-2126.4)	4061.7 (2564.1-5817)	181.85%	13.2 (9.62-20.46)	18.2 (13.31-26.94)	1.11 (0.98 to 1.24)
El Salvador	100.6 (64.1-146)	178.4 (108.4-257.9)	77.34%	10.22 (7.57-14.86)	10.51 (7.51-15.19)	0.1 (0.07 to 0.14)
Equatorial Guinea	4.3 (2.5-6.7)	14.6 (9-22.3)	239.53%	5.22 (3.63-8.56)	5.43 (4.11-8.41)	0.12 (0.09 to 0.15)
Eritrea	31.6 (18.1-50.5)	109.2 (63.4-170.3)	245.57%	5.89 (3.91-9.59)	8.05 (5.69-13.03)	1.06 (0.94 to 1.19)
Estonia	9.4 (5.5-14.9)	10.2 (5.9-16)	8.51%	2.28 (1.77-3.64)	3.13 (2.51-4.92)	1.11 (0.97 to 1.25)
Eswatini	7.2 (3.8-11.9)	12.9 (7.7-20.6)	373.61%	4.49 (3.18-7.74)	4.62 (3.52-7.61)	0.08 (-0.08 to 0.23)
Ethiopia	703.8 (339-1136.4)	1415.2 (809.7-2171.4)	101.08%	7.75 (4.92-12.93)	7 (5.09-10.83)	-0.32 (-0.41 to -0.22)
Fiji	3.8 (2.2-6)	5.3 (3.1-8.2)	39.47%	2.18 (1.65-3.52)	2.34 (1.74-3.67)	0.26 (0.21 to 0.31)
Finland	54 (32.4-81.8)	67 (39.5-101.4)	24.07%	3.77 (3.04-5.77)	5.28 (4.23-7.99)	1.18 (1.1 to 1.27)
France	1152.5 (677.9-1771.6)	822.2 (491.4-1281.9)	-28.66%	7.49 (5.78-11.22)	5.07 (4.13-7.5)	-1.36 (-1.57 to -1.16)
Gabon	7.9 (4.7-12.1)	23.6 (14.1-35.8)	198.73%	4.67 (3.2-7.61)	5.45 (4.06-8.58)	0.53 (0.5 to 0.56)
Gambia	3.4 (1.8-5.7)	11.9 (6.4-20.5)	250%	2.09 (1.45-3.78)	2.66 (2.01-4.64)	0.85 (0.67 to 1.04)
Georgia	53.1 (30.4-84.2)	33.4 (19.2-53.1)	-37.10%	3.99 (2.95-6.15)	3.42 (2.59-5.32)	-0.48 (-0.7 to -0.27)
Germany	1730.8 (1066.9-2572.9)	1545.3 (907.2-2364.6)	-72.93%	8.36 (6.55-12.34)	7.64 (6.05-11.64)	-0.31 (-0.36 to -0.26)
Ghana	63.5 (31.2-108.4)	173.3 (88.2-301.3)	172.91%	2.23 (1.45-4.18)	2.25 (1.62-3.97)	0.01 (-0.12 to 0.14)
Greece	114.6 (66.7-180.7)	96.5 (57.3-150.4)	-15.79%	4.31 (3.41-6.73)	3.49 (2.83-5.38)	-0.73 (-0.76 to -0.69)
Greenland	2.3 (1.6-3)	2.1 (1.5-2.8)	-8.70%	15.91 (12.61-21.97)	16.38 (12.75-22.37)	0.1 (0.06 to 0.14)
Grenada	0.6 (0.3-0.9)	0.8 (0.5-1.2)	33.33%	3.67 (2.75-5.7)	3 (2.26-4.63)	-0.7 (-0.78 to -0.63)
Guam	1.2 (0.7-1.8)	1.3 (0.8-2.1)	8.33%	3.7 (2.8-5.89)	3.38 (2.54-5.45)	-0.35 (-0.51 to -0.19)
Guatemala	239.1 (157.2-340.3)	715.1 (455.2-1013.2)	199.08%	17.14 (12.76-24.54)	17.22 (12.64-24.66)	0.05 (0 to 0.09)
Guinea	26.8 (12.4-46.5)	57.4 (28.5-97.6)	114.18%	2.3 (1.4-4.31)	2.3 (1.57-4.16)	0.05 (-0.18 to 0.29)
Guinea-Bissau	4.6 (2.2-8.2)	10.4 (5.1-17.6)	126.09%	2.47 (1.53-4.86)	2.52 (1.7-4.58)	0.06 (-0.01 to 0.13)
Guyana	9.2 (5.5-14.2)	12.2 (7.1-19.1)	32.61%	5.89 (4.32-9.31)	6.28 (4.71-9.87)	0.21 (0.16 to 0.27)
Haiti	51.9 (28.7-85.6)	145.1 (82.8-234.4)	-91.14%	4.18 (2.94-6.96)	4.75 (3.4-7.61)	0.45 (0.42 to 0.48)
Honduras	123.1 (78.4-182.7)	372.5 (233.8-557.4)	202.60%	15.9 (11.61-23.04)	16.41 (11.78-23.77)	0.11 (0.1 to 0.12)
Hungary	330.6 (196.5-490.3)	150.3 (91.9-232.5)	-54.54%	11.68 (8.88-17.61)	5.18 (3.9-7.82)	-2.77 (-2.93 to -2.62)
Iceland	0.9 (0.5-1.3)	0.9 (0.5-1.5)	0%	1.33 (1.09-2.05)	1.11 (0.92-1.78)	-0.6 (-0.65 to -0.56)
India	3581.2 (1588.1-5970.9)	12952.9 (6782.9-20910.8)	261.69%	2.06 (1.27-3.47)	3.68 (2.65-5.88)	1.94 (1.74 to 2.14)
Indonesia	3967.4 (1835.8-6517.4)	5197.5 (2873.6-7880.3)	31.01%	9.82 (6.16-16.19)	7 (4.99-10.66)	-1.15 (-1.37 to -0.93)
Iran (Islamic Republic of)	295.8 (155.8-458.5)	1061.3 (598.8-1611.5)	258.79%	3.05 (2.05-4.89)	4.17 (3.14-6.32)	1.07 (1.02 to 1.12)

Iraq	125.5 (72.6-190.1)	567.6 (344.6-813.5)	352.27%	4.45 (3.17-6.93)	5.85 (4.2-8.75)	0.95 (0.91 to 0.99)
Ireland	28.4 (17.5-42.7)	59 (35.1-90.1)	107.75%	3.29 (2.65-4.93)	4.29 (3.49-6.46)	0.9 (0.84 to 0.96)
Israel	52 (30.1-82.9)	116.3 (69.8-179.9)	123.65%	4.49 (3.51-6.95)	5.08 (4-7.68)	0.44 (0.39 to 0.48)
Italy	2718.4 (1847.9-3680.1)	1533 (977.7-2206.7)	-98.45%	17.99 (14.01-24.62)	8.79 (6.66-12.72)	-2.47 (-2.83 to -2.1)
Jamaica	9 (5.3-13.8)	13.9 (8.2-22)	54.44%	1.92 (1.44-3.01)	1.84 (1.48-2.87)	-0.15 (-0.24 to -0.05)
Japan	6264.5 (4034.5-8663.5)	5684.3 (4134.7-7428.7)	-9.26%	16.46 (12.33-22.69)	17.08 (13.65-22.46)	0.2 (-0.02 to 0.42)
Jordan	26.2 (15.9-40.2)	138.2 (84.6-199.7)	427.48%	4.6 (3.28-7.17)	5.28 (3.95-7.8)	0.47 (0.43 to 0.51)
Kazakhstan	149.3 (86.9-232.5)	645.4 (360.3-1027.7)	332.28%	4.1 (3.03-6.31)	12.61 (9.1-19.74)	3.97 (3.84 to 4.09)
Kenya	89.2 (45.6-144.9)	302.5 (157.7-493.6)	239.13%	2.49 (1.67-4.24)	2.85 (1.97-4.74)	0.51 (0.22 to 0.81)
Kiribati	0.7 (0.4-1.1)	1.2 (0.7-2)	71.43%	4.02 (2.73-7.04)	4.26 (3.18-7.22)	0.23 (0.09 to 0.36)
Kuwait	12.1 (7.5-18)	80.2 (50.5-119.5)	562.81%	3.55 (2.69-5.28)	5.06 (3.91-7.41)	1.25 (1.16 to 1.35)
Kyrgyzstan	60.6 (35.6-92)	167.7 (100.1-262)	176.73%	7.47 (5.59-11.56)	10.46 (7.99-15.68)	1.14 (0.9 to 1.39)
Lao People's Democratic Republic	43.4 (25.2-67.3)	113.2 (68.6-175.3)	160.83%	5.52 (4-8.77)	6.52 (5.01-10.11)	0.56 (0.5 to 0.61)
Latvia	16.7 (9.8-26.1)	16.2 (9.3-25.6)	-2.99%	2.4 (1.91-3.81)	3.43 (2.66-5.46)	1.24 (1.14 to 1.35)
Lebanon	25.2 (14.8-38.7)	78.4 (47.9-118)	211.11%	3.64 (2.58-5.64)	5.34 (4.05-7.97)	1.41 (1.2 to 1.61)
Lesotho	15.1 (7.8-25.1)	23.5 (12.7-37.5)	55.63%	4.25 (2.86-7.51)	4.53 (3.24-7.6)	0.19 (0.12 to 0.27)
Liberia	6.8 (3-12.7)	25 (13-42.2)	267.65%	1.93 (1.31-3.82)	2.33 (1.73-4.15)	0.65 (0.56 to 0.73)
Libya	27.3 (16.5-40.8)	110 (67.1-165.6)	302.93%	4.5 (3.13-6.99)	5.17 (3.91-7.8)	0.52 (0.28 to 0.77)
Lithuania	22.8 (13.4-35.1)	31.3 (17.9-48.5)	37.28%	2.4 (1.88-3.73)	4.84 (3.83-7.75)	2.48 (2.27 to 2.7)
Luxembourg	9.6 (5.8-14.7)	11.6 (7-18.1)	20.83%	9.08 (7.27-13.52)	6.53 (5.22-9.87)	-1.16 (-1.24 to -1.07)
Madagascar	113.7 (62.3-182.7)	390.8 (228.3-597.7)	243.71%	5.49 (3.76-9.42)	7.11 (5.17-11.09)	0.9 (0.83 to 0.97)
Malawi	93 (53.5-149.5)	185.1 (108.9-289.1)	99.03%	5.65 (3.8-9.46)	5.39 (3.92-8.47)	-0.17 (-0.23 to -0.11)
Malaysia	116.5 (70.3-177)	319.7 (200.7-479.3)	174.42%	3.13 (2.26-4.87)	4.03 (3-6.02)	0.88 (0.83 to 0.92)
Maldives	1.2 (0.7-1.8)	4.5 (2.7-7)	3150%	3.43 (2.51-5.3)	3.8 (2.94-5.83)	0.34 (0.29 to 0.39)
Mali	37.2 (16.1-68.6)	105.2 (56.8-179.4)	182.80%	2.38 (1.48-4.57)	2.77 (1.92-4.91)	0.5 (0.31 to 0.69)
Malta	4.4 (2.7-6.7)	3.6 (2.2-5.5)	-18.18%	4.13 (3.39-6.06)	3.26 (2.6-4.89)	-0.8 (-0.87 to -0.73)
Marshall Islands	0.3 (0.2-0.5)	0.5 (0.3-0.8)	112700%	3.59 (2.42-5.97)	3.63 (2.63-5.89)	-0.01 (-0.13 to 0.12)
Mauritania	8.4 (4.2-14.3)	19.3 (10.5-32.2)	53.57%	2.3 (1.64-4.18)	2.33 (1.74-3.94)	0.06 (-0.01 to 0.13)
Mauritius	13.5 (8.4-19.9)	14.4 (8.9-21.5)	6.67%	4.8 (3.62-7.24)	4.18 (3.22-6.25)	-0.52 (-0.64 to -0.39)
Mexico	3277.9 (2095.1-4613.6)	5714.5 (3641.5-8076.5)	74.33%	19.94 (14.23-28.25)	16.14 (11.94-23.4)	-0.68 (-0.88 to -0.48)
Micronesia (Federated States of)	0.6 (0.4-1)	0.9 (0.5-1.5)	50%	3.45 (2.49-5.84)	3.83 (2.89-6.26)	0.33 (0.23 to 0.43)
Monaco	0.5 (0.3-0.8)	0.7 (0.4-1.1)	40%	6.08 (4.72-9.16)	7.77 (6.06-11.77)	0.82 (0.74 to 0.9)
Mongolia	37.1 (21.1-60.4)	131.4 (67.2-220.5)	254.18%	10.35 (7.11-17.22)	13.32 (9.14-22.65)	0.9 (0.83 to 0.97)
Montenegro	3 (1.8-4.6)	3.4 (2-5.2)	13.33%	1.93 (1.56-2.97)	2.07 (1.66-3.14)	0.31 (0.15 to 0.48)
Morocco	170.9 (98.2-266.8)	497 (301.1-722.6)	190.81%	3.47 (2.47-5.51)	5.1 (3.67-7.53)	1.35 (1.27 to 1.44)
Mozambique	90.6 (50.3-148.8)	210.2 (116.6-329)	132.01%	3.56 (2.47-5.98)	3.88 (2.84-6.24)	0.29 (0.23 to 0.35)
Myanmar	634.8 (384.9-966.3)	1513.5 (904.6-2313.7)	138.42%	7.34 (5.2-11.46)	10.02 (7.74-15.27)	1.05 (0.91 to 1.2)
Namibia	11.1 (6.4-17.8)	25.5 (14.6-40.2)	129.73%	4.02 (2.86-6.79)	4.33 (3.14-6.84)	0.22 (0.15 to 0.29)
Nauru	0.1 (0-0.1)	0.1 (0.1-0.2)	0%	2.83 (2.08-4.77)	3.72 (2.84-6.03)	0.93 (0.87 to 0.98)
Nepal	52.8 (28.6-88.6)	174.6 (95.6-303.6)	230.68%	1.43 (1.06-2.49)	2.24 (1.7-3.89)	1.54 (1.4 to 1.69)
Netherlands	183.4 (110.3-285.2)	160.5 (95.1-246.8)	-12.49%	4.39 (3.53-6.69)	3.85 (3-5.99)	-0.45 (-0.49 to -0.4)
New Zealand	32.6 (21.2-45.5)	42.8 (30.3-58)	31.29%	3.6 (2.7-5.13)	3.81 (3.05-5.3)	0.2 (0.17 to 0.22)
Nicaragua	56.5 (36.7-78.8)	167.4 (105.6-246.3)	196.28%	8.98 (6.75-12.93)	10.4 (7.72-15.07)	0.52 (0.41 to 0.63)
Niger	37.2 (16.9-66.2)	106.6 (55.6-182.8)	186.56%	2.8 (1.7-5.3)	2.97 (2.04-5.27)	0.18 (0.1 to 0.27)
Nigeria	287 (126.8-492.9)	1086.2 (530.6-1768.4)	278.47%	1.95 (1.15-3.4)	2.53 (1.72-4.21)	0.9 (0.72 to 1.09)
Niue	0 (0-0)	0 (0-0)	NaN%	2.6 (1.96-4.13)	3.24 (2.38-5.19)	0.76 (0.59 to 0.93)

North Macedonia	17.9 (10.4-27.6)	23.5 (14.2-37.1)	31.28%	3.49 (2.62-5.42)	3.85 (2.99-5.8)	0.34 (0.26 to 0.42)
Northern Mariana Islands	0.7 (0.4-1.2)	0.5 (0.3-0.9)	-28.57%	6.02 (4.27-9.73)	5.55 (4.14-9.07)	-0.3 (-0.44 to -0.16)
Norway	37.9 (24-55)	42.2 (26.9-60.8)	2734.56%	3.4 (2.62-4.97)	3.15 (2.47-4.68)	-0.15 (-0.43 to 0.13)
Oman	16.7 (9.9-25.2)	71 (44.7-103.6)	325.15%	6.65 (4.63-10.23)	8.28 (6.02-12.07)	0.76 (0.59 to 0.93)
Pakistan	2244.6 (1021.4-3598.1)	5519.4 (2717.2-8876)	145.90%	11.66 (7.14-19.31)	11.85 (7.45-19.24)	0.06 (0.01 to 0.11)
Palau	0.1 (0.1-0.2)	0.2 (0.1-0.3)	100%	2.85 (2.12-4.58)	3.36 (2.58-5.29)	0.56 (0.42 to 0.71)
Palestine	14.6 (9-22.1)	61.8 (39.2-87.7)	323.29%	4.71 (3.43-7.17)	6.13 (4.49-9.05)	0.88 (0.65 to 1.11)
Panama	32.4 (20.6-47.9)	67.1 (41-98.8)	107.10%	6.56 (4.89-9.42)	6.36 (4.72-9.29)	-0.06 (-0.15 to 0.02)
Papua New Guinea	4.3 (2.2-7.5)	13.8 (6.7-23.2)	220.93%	0.52 (0.36-0.92)	0.59 (0.41-1.04)	0.43 (0.4 to 0.47)
Paraguay	29.1 (18.8-42.2)	62.2 (39-90.4)	113.75%	3.68 (2.84-5.37)	3.64 (2.75-5.42)	-0.02 (-0.08 to 0.04)
Peru	111.4 (62.2-177.9)	251.2 (139.4-423.2)	125.49%	2.65 (2.02-4.39)	2.74 (2.07-4.53)	0.1 (0 to 0.21)
Philippines	372.3 (217.4-566.5)	1202 (734.3-1781.1)	222.86%	2.93 (2.06-4.54)	4.54 (3.25-6.73)	1.55 (1.34 to 1.76)
Poland	441.9 (265.9-657)	471.3 (282.2-707.2)	6.65%	4.41 (3.3-6.48)	4.31 (3.35-6.48)	-0.05 (-0.14 to 0.04)
Portugal	207.6 (125.7-317.8)	114 (67-176.5)	-45.09%	8.07 (6.51-12)	3.8 (2.96-5.68)	-2.6 (-2.99 to -2.21)
Puerto Rico	50.5 (30.4-77)	37.6 (21.8-58.6)	-25.54%	5.27 (3.91-8.18)	3.98 (2.88-6.08)	-0.96 (-1.05 to -0.87)
Qatar	3.8 (2.2-6)	41 (24.6-62.3)	978.95%	6 (4.2-9.71)	8.42 (6.24-12.72)	1.21 (1 to 1.42)
Republic of Korea	584.1 (366.4-878)	468.6 (287.8-714.6)	162.46%	5.35 (4.03-8.09)	3.06 (2.52-4.67)	-1.97 (-2.22 to -1.72)
Republic of Moldova	246.4 (145.6-393.8)	197.7 (110.8-323.3)	-19.76%	22.31 (16.27-35.3)	17.87 (13.16-27.8)	-0.75 (-0.92 to -0.57)
Romania	718.7 (434.4-1102.8)	680.2 (396.8-1043.6)	-5.36%	12.45 (9.43-18.61)	11.79 (8.58-17.51)	-0.19 (-0.32 to -0.05)
Russian Federation	1162.2 (692.5-1778)	2308.5 (1304.9-3576.1)	98.63%	2.97 (2.33-4.54)	5.54 (4.11-8.77)	2.19 (2.13 to 2.25)
Rwanda	134.5 (82.1-204.4)	290.1 (184.7-424.7)	115.69%	10.7 (7.19-17.01)	10.55 (7.81-16.24)	-0.05 (-0.08 to -0.03)
Saint Kitts and Nevis	0.4 (0.2-0.5)	0.6 (0.3-0.9)	50%	4.81 (3.7-7.46)	3.29 (2.48-5.08)	-1.33 (-1.4 to -1.26)
Saint Lucia	1.4 (0.8-2.2)	2.3 (1.3-3.5)	64.29%	5.53 (4.21-8.69)	4.31 (3.31-6.52)	-0.87 (-0.92 to -0.81)
Saint Vincent and the Grenadines	0.5 (0.3-0.8)	0.7 (0.4-1.1)	40%	2.68 (1.98-4.15)	2.38 (1.83-3.71)	-0.41 (-0.49 to -0.33)
Samoa	0.9 (0.5-1.5)	1.6 (0.9-2.5)	11422.22%	3.39 (2.5-5.47)	3.68 (2.68-5.65)	0.27 (0.24 to 0.31)
San Marino	0.6 (0.4-1)	1.1 (0.7-1.6)	83.33%	11.1 (8.7-16.51)	11.27 (8.55-17.06)	0.03 (-0.04 to 0.1)
Sao Tome and Principe	0.5 (0.3-0.8)	1.3 (0.7-2.2)	160%	2.65 (1.76-4.77)	2.82 (2.12-4.89)	0.18 (-0.01 to 0.37)
Saudi Arabia	197.7 (124-282.1)	1037.3 (653.8-1508.7)	424.68%	8.53 (6.01-12.75)	10.36 (7.7-15.14)	0.67 (0.44 to 0.9)
Senegal	27.3 (13.5-47.8)	73.2 (38.8-124.1)	168.13%	2.1 (1.4-3.89)	2.36 (1.66-4.07)	0.43 (0.37 to 0.49)
Serbia	88.8 (51.6-138.3)	75.4 (45.2-116.9)	-15.09%	3.65 (2.85-5.55)	3.3 (2.54-5.14)	-0.32 (-0.4 to -0.25)
Seychelles	0.8 (0.5-1.2)	1.8 (1.1-2.7)	125%	5.96 (4.47-8.82)	6.48 (4.87-9.66)	0.28 (0.23 to 0.33)
Sierra Leone	13.3 (5.8-25.2)	37.9 (19.5-65.2)	184.96%	1.92 (1.21-3.65)	2.24 (1.61-3.98)	0.6 (0.42 to 0.79)
Singapore	16.7 (9.8-26.6)	28.9 (17.4-45.2)	73.05%	1.85 (1.41-2.89)	1.58 (1.27-2.39)	-0.54 (-0.59 to -0.5)
Slovakia	110.1 (67.6-169.3)	119.8 (72.3-181)	8.81%	7.79 (6.07-11.64)	7.5 (5.76-11.1)	-0.13 (-0.21 to -0.06)
Slovenia	59.4 (36.3-88)	33.4 (20.6-50.7)	-43.77%	11.28 (8.76-16.27)	6.04 (4.81-8.96)	-2.15 (-2.34 to -1.96)
Solomon Islands	2.7 (1.5-4.6)	7.3 (4.1-12)	170.37%	4.19 (3.07-7.39)	4.71 (3.53-8.12)	0.42 (0.34 to 0.49)
Somalia	114.1 (51.3-199.5)	306.8 (162.1-491.9)	168.89%	8.53 (5.6-14.88)	8.37 (5.86-14.29)	-0.06 (-0.11 to -0.02)
South Africa	297.8 (149.1-487.7)	462.7 (247.9-734.6)	55.37%	3.45 (2.23-5.7)	3.04 (2.14-4.68)	-0.44 (-0.52 to -0.37)
South Sudan	52.1 (29.4-83.2)	108.4 (60-175.1)	108.06%	5.61 (3.9-9.34)	5.83 (4.13-9.52)	0.15 (0.08 to 0.21)
Spain	1139 (754.9-1618.2)	1074.3 (699.4-1551)	35.67%	11.87 (9.26-17.2)	8.09 (6.7-11.65)	-1.32 (-1.38 to -1.26)
Sri Lanka	178.5 (111.2-260.9)	265.5 (165.4-398)	-99.72%	4.25 (3.24-6.35)	4.43 (3.57-6.69)	0.14 (0.07 to 0.22)
Sudan	119.8 (65.5-192)	415.5 (247.7-623.1)	246.83%	3.41 (2.3-5.53)	4.83 (3.62-7.46)	1.22 (1.1 to 1.34)
Suriname	3.8 (2.3-6)	6.7 (3.8-10.6)	76.32%	4.73 (3.51-7.36)	4.34 (3.23-6.79)	-0.31 (-0.38 to -0.24)
Sweden	75.8 (49.2-107.9)	85.5 (54.7-124.9)	12.80%	3.31 (2.6-4.9)	3.45 (2.7-5.08)	0.16 (0.1 to 0.22)
Switzerland	84.8 (51.3-129.3)	100.1 (60.3-155.9)	18.04%	4.39 (3.51-6.63)	4.31 (3.49-6.61)	-0.11 (-0.26 to 0.04)
Syrian Arab Republic	95.1 (54.8-146.1)	239.6 (146.5-353.7)	151.95%	4.91 (3.63-7.75)	6.01 (4.47-8.98)	0.71 (0.66 to 0.76)

Taiwan (Province of China)	285.2 (171.9-441.4)	329.1 (180.9-533.2)	15.39%	5.96 (4.44-9.39)	4.44 (3.25-6.77)	-1 (-1.09 to -0.91)
Tajikistan	51.4 (30.7-78.6)	207 (115.8-336.6)	302.72%	6.09 (4.56-9.66)	9.58 (7.17-15.28)	1.59 (1.54 to 1.65)
Thailand	931.2 (590.1-1354.1)	1456.4 (866.7-2222.4)	56.40%	6.54 (4.81-9.8)	6.69 (5.12-10.12)	0.07 (-0.01 to 0.15)
Timor-Leste	7.8 (4.7-12.1)	17.3 (10.6-26.1)	121.79%	5.09 (3.77-8)	6.86 (5.24-10.54)	1.09 (1.01 to 1.17)
Togo	14.6 (7-25)	39.9 (20.9-69.3)	173.29%	2.23 (1.46-4.11)	2.17 (1.57-3.82)	-0.12 (-0.19 to -0.04)
Tokelau	0 (0-0)	0 (0-0)	0%	2.59 (1.87-4.33)	2.57 (2.01-4.14)	-0.03 (-0.07 to 0.02)
Tonga	0.8 (0.4-1.2)	1.1 (0.6-1.7)	37.50%	4.31 (3.13-6.86)	4.71 (3.43-7.63)	0.27 (0.16 to 0.38)
Trinidad and Tobago	9.2 (5.6-14)	11 (6.2-17)	19.57%	3.31 (2.44-5.19)	2.85 (2.2-4.4)	-0.52 (-0.56 to -0.47)
Tunisia	74.7 (44.8-109)	198.6 (121.4-292.8)	165.86%	4.74 (3.29-7.21)	5.8 (4.35-8.55)	0.72 (0.61 to 0.82)
Turkey	263.5 (153.1-412.7)	789.6 (457.1-1248.9)	199.66%	2.2 (1.55-3.48)	3.34 (2.53-5.25)	1.52 (1.35 to 1.69)
Turkmenistan	50.3 (30.1-78.3)	129.9 (73.7-203.9)	158.25%	7.67 (5.81-12.11)	10.34 (8.11-16.68)	1.07 (0.97 to 1.17)
Tuvalu	0.1 (0-0.1)	0.1 (0-0.1)	0%	2.59 (1.91-4.26)	3.07 (2.34-4.89)	0.59 (0.56 to 0.62)
Uganda	122.7 (70.6-194.2)	349.1 (202.4-537.4)	184.52%	4.53 (3.18-7.57)	4.85 (3.62-7.81)	0.22 (0.09 to 0.35)
Ukraine	392.1 (227.9-602.6)	516.7 (238.7-863.3)	31.78%	2.89 (2.16-4.53)	4.28 (3.14-7.32)	1.37 (1.28 to 1.47)
United Arab Emirates	9.2 (5.4-14.1)	109.1 (64.8-168.1)	1085.87%	3.68 (2.75-5.8)	5.13 (3.78-7.65)	1.22 (0.97 to 1.46)
United Kingdom	208.7 (137.2-299.5)	401.9 (283.2-546)	92.57%	1.4 (1.11-2.02)	2.4 (1.92-3.35)	1.9 (1.77 to 2.03)
United Republic of Tanzania	206.7 (120.6-323.7)	589 (342.1-924.7)	184.95%	4.8 (3.18-7.9)	5.22 (3.88-8.35)	0.27 (0.17 to 0.37)
United States of America	9413.5 (6686.1-12834.5)	17787.1 (13732.4-22376.2)	88.95%	13.15 (10.1-18.14)	21.61 (17.79-27.48)	1.7 (1.53 to 1.88)
United States Virgin Islands	1.3 (0.7-2.1)	1.1 (0.6-1.8)	-15.38%	4.14 (3.18-6.84)	4.17 (3.14-6.47)	0.02 (-0.03 to 0.06)
Uruguay	37.7 (24.3-53.7)	40.8 (25.8-59.6)	8.22%	4.99 (3.82-7.13)	4.52 (3.51-6.71)	-0.29 (-0.49 to -0.09)
Uzbekistan	308.7 (181.3-476.2)	1126 (666-1760.3)	-13.99%	8.91 (6.72-14.34)	12.9 (9.8-19.97)	1.3 (1.24 to 1.36)
Vanuatu	1.3 (0.7-2.1)	3 (1.7-4.7)	130.77%	4.58 (3.37-7.61)	4.63 (3.48-7.63)	0.02 (-0.03 to 0.06)
Venezuela (Bolivarian Republic of)	252.1 (165.8-358)	389.3 (246.4-566.5)	54.42%	6.57 (4.9-9.45)	4.92 (3.73-7.13)	-1 (-1.1 to -0.89)
Viet Nam	566.4 (354.2-858.6)	1507.5 (921.3-2268.6)	-99.72%	4.32 (3.22-6.57)	5.27 (4.09-7.85)	0.67 (0.53 to 0.81)
Yemen	94 (52.4-148.3)	365.8 (220.5-545.6)	289.15%	4.75 (3.19-7.55)	5.91 (4.12-8.92)	0.77 (0.71 to 0.83)
Zambia	91.6 (51.6-145.9)	259.9 (153.7-399.6)	183.73%	7 (4.95-11.78)	7.43 (5.35-12.04)	0.21 (0.11 to 0.32)
Zimbabwe	62.5 (37.7-95.2)	141.6 (79.7-223.4)	126.56%	3.47 (2.49-5.52)	4.22 (3.16-7.02)	0.69 (0.6 to 0.77)

Abbreviations: ASR, age-standardized rate; AAPC, average annual percent change; UI, uncertainty interval; CI, confidence interval.

Figure S1. Temporal change in the relative proportion of AHC incidence across seven age groups from 1990 to 2019. Abbreviations: AHC, acute hepatitis C.

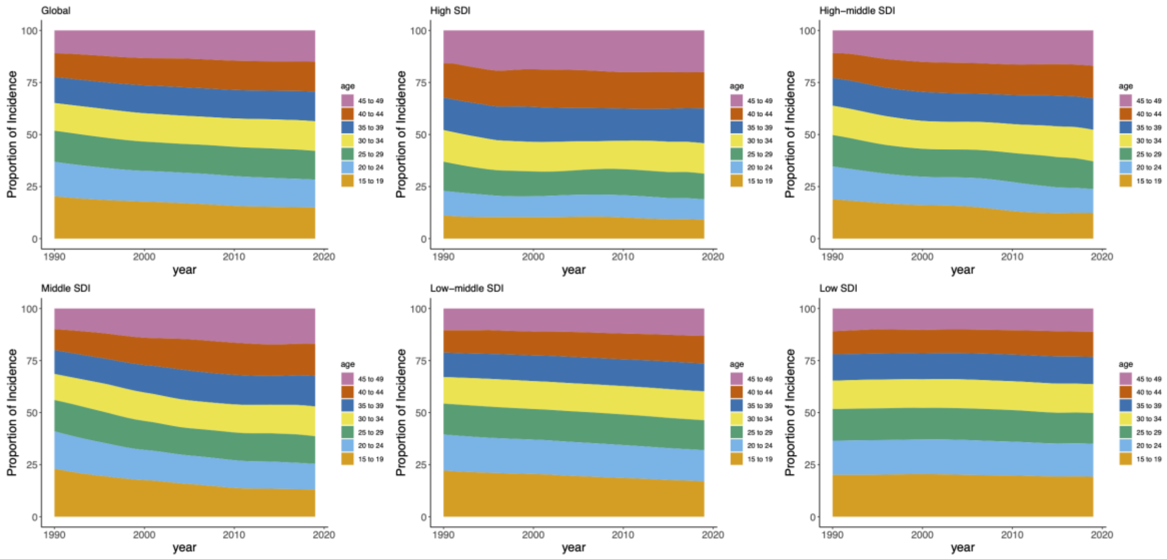

Figure S2. Temporal change in the relative proportion of HCV-related cirrhosis incidence across seven age groups from 1990 to 2019. Abbreviations: HCV, hepatitis C virus.

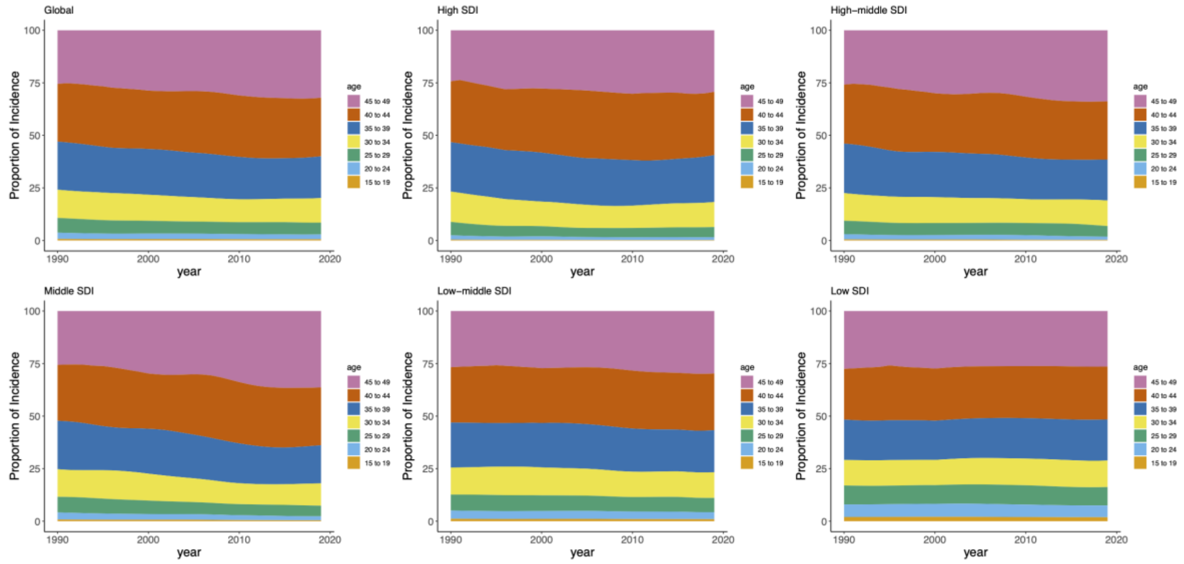

Figure S3. The correlation between SDI and ASIR of AHC (Panel A) and HCV-related cirrhosis (Panel B) in 2019, and the corresponding AAPC (Panel C and Panel D) from 1990 to 2019. Abbreviations: ASIR, age-standardized incidence rate; AAPC, average annual percent change; AHC, acute hepatitis C; HCV, hepatitis C virus.

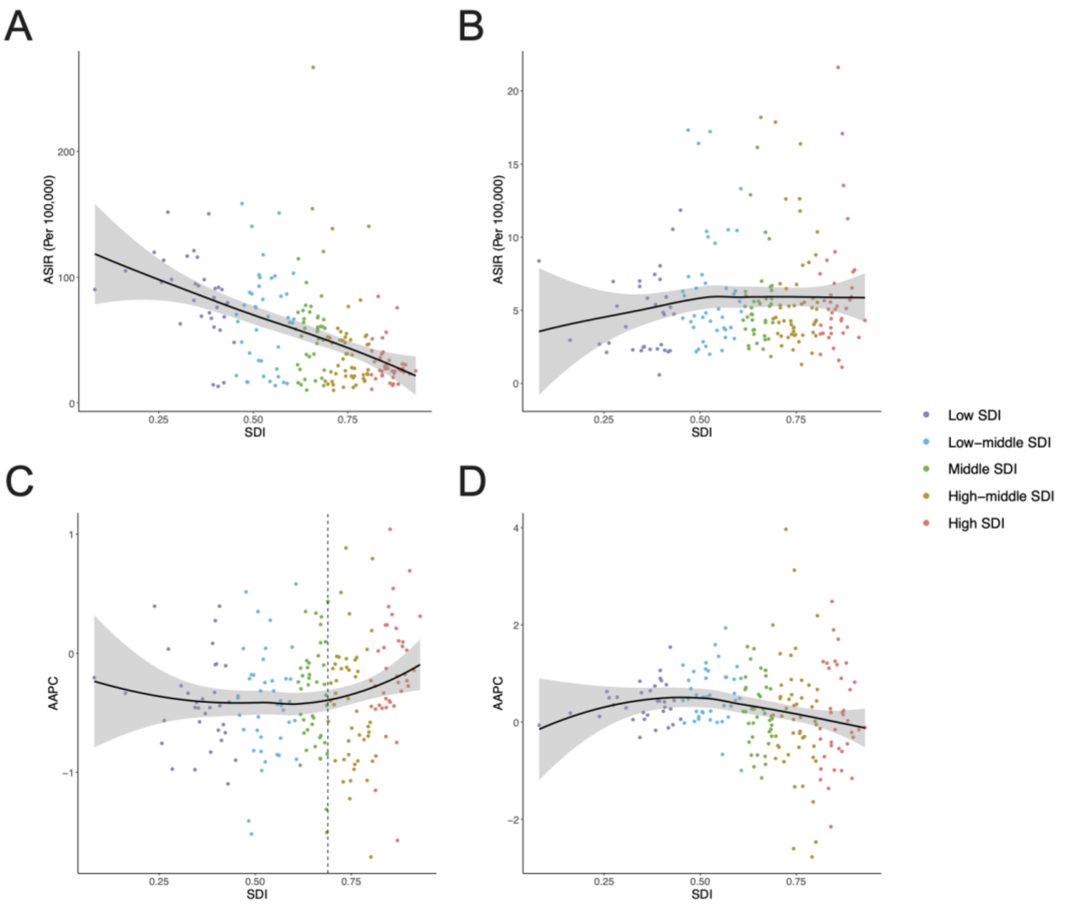

Figure S4. The correlation between AAPC and ASIR of AHC (Panel A) and HCV-related cirrhosis (Panel B) in 1990. The size of the dots represents the number of cases in 1990. Abbreviations: ASIR, age-standardized incidence rate; AAPC, average annual percent change; AHC, acute hepatitis C; HCV, hepatitis C virus.

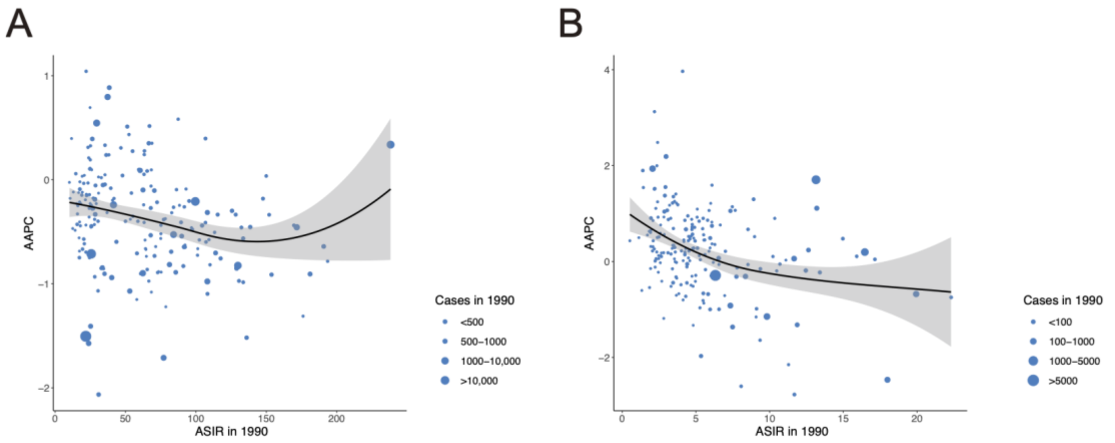

Supplement: Online Supplementary Document [file jogh-14-04077-s001.pdf]
